# Microfluidic and Nanofluidic Intracellular Delivery

**DOI:** 10.1002/advs.202004595

**Published:** 2021-06-06

**Authors:** Jeongsoo Hur, Aram J. Chung

**Affiliations:** ^1^ School of Biomedical Engineering Korea University Seoul 02841 Republic of Korea; ^2^ School of Biomedical Engineering Interdisciplinary Program in Precision Public Health Korea University Seoul 02841 Republic of Korea

**Keywords:** cell transfection, gene delivery, intracellular delivery, microfluidics, nanofluidics

## Abstract

Innate cell function can be artificially engineered and reprogrammed by introducing biomolecules, such as DNAs, RNAs, plasmid DNAs, proteins, or nanomaterials, into the cytosol or nucleus. This process of delivering exogenous cargos into living cells is referred to as intracellular delivery. For instance, clustered regularly interspaced short palindromic repeats (CRISPR)‐Cas9 gene editing begins with internalizing Cas9 protein and guide RNA into cells, and chimeric antigen receptor‐T (CAR‐T) cells are prepared by delivering CAR genes into T lymphocytes for cancer immunotherapies. To deliver external biomolecules into cells, tools, including viral vectors, and electroporation have been traditionally used; however, they are suboptimal for achieving high levels of intracellular delivery while preserving cell viability, phenotype, and function. Notably, as emerging solutions, microfluidic and nanofluidic approaches have shown remarkable potential for addressing this open challenge. This review provides an overview of recent advances in microfluidic and nanofluidic intracellular delivery strategies and discusses new opportunities and challenges for clinical applications. Furthermore, key considerations for future efforts to develop microfluidics‐ and nanofluidics‐enabled next‐generation intracellular delivery platforms are outlined.

## Graphical abstract

This review provides an overview of recent advances in microfluidic and nanofluidic intracellular delivery strategies, enabling cellular engineering and cell therapy. New opportunities and challenges from micro(nano)fluidic intracellular delivery are highlighted, and key considerations on the establishment of microfluidics‐ and nanofluidics‐enabled next‐generation intracellular delivery platforms are also discussed.

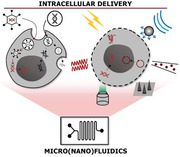

## 1 Introduction

**Figure 1:**
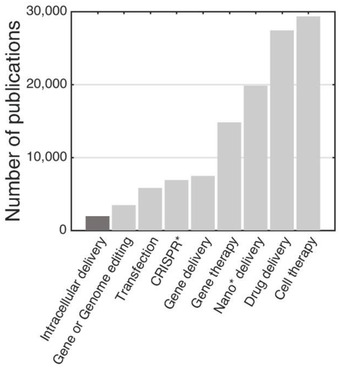
Publication analysis. The bar graph represents the number of publications containing the given keywords according to the ISI Web of Knowledge, retrieved in November 2020.

**Figure 2:**
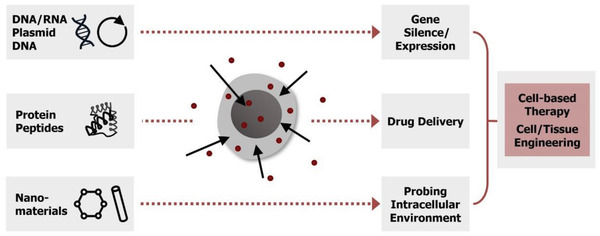
Motivations for intracellular delivery. Intracellular delivery of different external cargos into cells and the potential outcomes and applications. Red dots indicate the target material.

Using the keywords “gene or genomic editing,” “transfection,” “CRISPR*,” “gene delivery,” “nano* delivery,” “gene therapy,” “drug delivery,” and “cell therapy,” more than 110 000 articles were retrieved from the Web of Knowledge provided by Thomson Reuters, in November 2020 (**Figure** **1**). One common goal associated with these keywords is to engineer cell functions. To artificially alter specific cell functions in a desired manner, biomolecules such as DNAs, RNAs, plasmid DNAs, proteins, or nanomaterials, including gold, iron oxide, silica, and polymeric nanoparticles, are generally internalized into cells. This process of delivering exogenous cargos into living cells is known as “intracellular delivery.” The term has not been extensively adopted in the field (Figure 1), although it refers to all relevant steps associated with the internalization of external cargo(s) into the cytosol or nucleus. For example, chimeric antigen receptor (CAR) transgenes are delivered into the T lymphocytes of patients to generate CAR‐T cells that recognize and effectively kill tumors, demonstrating definitive evidence of clinical effectiveness (three CD19‐directed CAR‐T cell products—Kymriah™, Yescarta™, and Tecartus™—have been approved by the FDA).^[^ ^1^, ^2^ ^]^ For stem cell therapy, mature and fully differentiated cells can be reprogrammed into a pluripotent state by delivering pluripotency‐associated transcription factors, such as Oct4, Sox2, Klf4, and c‐Myc, and induced pluripotent stem cells (iPSCs) have offered unlimited promises and opportunities for treating degenerative diseases and cancer, and for studying disease pathology and drug screening.^[^ ^3^, ^4^ ^]^ Regarding genome editing, transcription activator‐like effector nucleases (TALEN) and clustered regularly interspaced short palindromic repeats (CRISPR)‐Cas9 gene editing systems, which have become indispensable tools that target gene knock‐in or knock‐out, can be accomplished by internalizing genetic elements into cells, thereby expanding its application in human disease therapy.^[^ ^5^ ^]^ Thus, intracellular delivery of external nano/biomaterial can be considered one of the fundamental steps and a starting point, enabling cellular engineering (**Figure** **2**).

**Figure 3:**
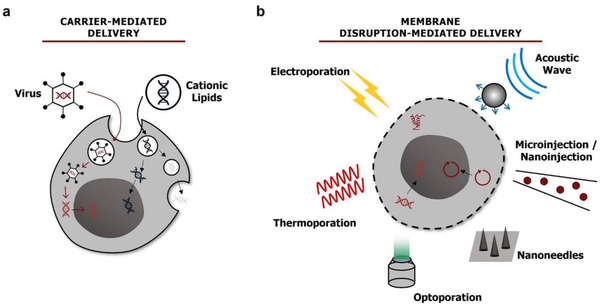
Two roads for the intracellular delivery of external cargos. a) Viral and nonviral carriers for intracellular delivery through endocytic, fusion, and infection pathways. b) Membrane disruption through electrical, thermal, optical, or acoustic energy or micro‐ or nanoscale conduit for exogenous cargo delivery.

Intracellular delivery of a cargo to the site of action (e.g., cytoplasm, nucleus, and subcellular organelles) with high specificity and subcellular level resolution guarantees successful and effective engineering of cell function. To realize the internalization of foreign biomolecules inside living cells, a number of methods have been proposed. More details can be found in subsequent sections of this review; they are commonly categorized into 1) carrier‐mediated and 2) membrane disruption‐based methods, as shown in **Figure** **3**. Carriers, such as viral vectors, lipids, polymers, liposomes, exosomes, cell‐penetrating peptides, and cell ghosts, are popular transport vehicles that deliver encapsulated cargo(s) into cells using their pathways (e.g., endocytic, fusion, and infection pathways).^[^ ^6^ ^]^ For example, viral vectors, such as lentivirus, retrovirus, herpes virus, and adeno‐associated virus (AAV), have been extensively used for nuclear acid transfection, and have shown successful clinical outcomes (e.g., gene therapies).^[^ ^7^ ^]^ Lipofection using cationic lipids is another prime example of a nonviral carrier‐mediated intracellular delivery method that delivers external cargos into cells via endocytosis. Alternatively, membrane disruption‐based techniques are based on applying external (electrical, thermal, optical, or mechanical) energy to cells to physically open the cellular membrane; by this means, external cargos dispersed in buffer solution can be internalized through the created discontinuities. Most popularly, electroporation is adopted in the laboratory, and its potential for clinical applications is being explored (e.g., NCT03608618).

One fundamental question for researchers who require intracellular delivery is, “Are you satisfied with your current choice of delivery method?” As mentioned above, there is a wide range of options, platforms, kits, techniques, and protocols designed for intracellular delivery. However, most of them suffer from at least one of the following issues: low and inconsistent delivery efficiency, cytotoxicity, low scalability, high cost, complexity in preparation, operational difficulty, loss of cell function and phenotype after delivery, and undesirable genotoxicity, mutagenesis, and immunogenicity. Furthermore, such issues become even more critical when the techniques are applied to primary cells, including stem and immune cells, toward ex vivo applications (e.g., cell therapy). It is imperative to note that an ideal method using either carrier‐mediated or membrane disruption‐mediated strategy should provide a high level of delivery, surpassing the existing gold standards such as viral transduction, lipofection, and electroporation. Moreover, the approach should not be restricted by cell type (e.g., suspension and adherent cells) and cargo characteristics (e.g., size, polarity, and morphology), and it should be applicable to hard‐to‐transfect primary cells. Additionally, the process must be non‐ or minimally invasive (i.e., high viability and maintenance of cell functionality after delivery), scalable (i.e., high throughput), current good manufacturing practice (cGMP) compliant, robust, dose controllable, cost‐effective, and easy to operate. However, a universal delivery method that meets all the aforementioned characteristics is yet to be identified (see **Table** **1**).

**Table 1:** Current bench‐top techniques and desired features expected from an ideal approach for intracellular delivery

Technologies	Efficiency	Nanoparticle delivery (>100 nm)	Primary cell applicability	Viability	Scalability (per run)	Cost
Electroporation	Medium to high (depends on cell and cargo type)	Δ (high Stokes drag)	Δ (low viability and functionality concern)	Low to high (depends on cell type)	10^4^ cells per run^a)^ ≈10^6^ cells per run^b)^	$10k^a)^ to 100k^b)^
Microinjection	Theoretically high^[^ ^219^ ^]^	O	O	Low to high (depends on cell type)	100 cells h^−1[^ ^80^ ^]^	$10k^c)^ (injector only)
Viral transduction	High but limited in DNA size^d)^	X (packaging failure)	O	Mutagensis concern	High to low (depends on viral amount)	High (preparation)
Lipofection	Low to high (depends on cell and cargo type)	X (packaging failure)	X (low efficiency for suspension cells)	Medium to high (depends on cell type)	High to low (depends on reagent amount)	$1k/50 tests^e)^
Ideal microfluidic method	Always high	O	O	Always high	High	Low

^a)^ Capillary electroporation (Neon transfection system);

^b)^ Cuvette electroporation (Lonza Nucleofector system);

^c)^ FemtoJet 4i model (Eppendorf);

^d)^ DNA size <5 kbp for AAV vector and <10 kbp for lentiviral vector^[^ ^220^ ^]^;

^e)^ Using lipofectamine 3000 for a test using 60 mm culture dish.

To tackle this open challenge, micro/nanotechnology‐enabled solutions have been substantially investigated.^[^ ^8^, ^9^ ^]^ Notably, as one of the solutions, microfluidic intracellular delivery approaches have shown unprecedented potential.^[^ ^10^, ^11^, ^12^, ^13^, ^14^ ^]^ The key benefits of using microfluidics can be understood from two standpoints: 1) controllability and 2) reduction in scale. Because of the small channel footprint and low fluid velocity, the associated Reynolds number (*Re*), a nondimensional parameter describing the ratio of the inertial force to the viscous force, becomes close to zero (see **Table** **2**). A low *Re* implies that fluid flow in microchannels is approximated by laminar flow, which inherently allows high spatiotemporal flow control. Thus, the subcellular level flow controllability enables a high level of precise cell manipulation, which consequently allows effective intracellular delivery. Other major benefits include a substantial reduction in sample and reagent volume due to a reduction in scale. Often, patient‐driven primary cells and sophisticatedly designed cargos are difficult to prepare in large quantities, and microfluidic integration could be a convenient approach. However, this does not necessarily mean that high throughput processing is unavailable because extremely large numbers of cells (i.e., large fluid volume) can be processed as well. Although more details are provided below, there are microfluidic platforms with the capability of processing cells at a rate of 10^6^–10^7^ cells min^−1^ per channel.^[^ ^15^ ^]^ Broadly speaking, cell‐based therapies require ≈10^8^–10^9^ cells per treatment;^[^ ^16^ ^]^ therefore, with channel parallelization and high‐speed operation, micro‐ and nanofluidic platforms could conveniently meet the required throughput. In addition, miniaturization inherently permits the integration of microfluidics with other modalities, opening new possibilities for fully automated systems. It should be mentioned that automation eliminates user‐based bias by bypassing unnecessary human handling steps, thereby preserving sample integrity.^[^ ^17^ ^]^ This automation process intrinsically allows robustness and repeatability in the cell handling process to standardize the intracellular delivery process.

**Table 2:** Glossary.^[^ ^221^, ^222^, ^223^ ^]^

Convection	Transfer of mass due to the bulk movement of molecules within fluids utilized for several intracellular delivery techniques as a driving force for transporting macromolecules. For convective intracellular delivery, an external force is required for the internalization of a cargo.
Delivery (transfection) efficiency	A ratio (%) of cells with successful cargo delivery to total cells. In intracellular delivery, this parameter is used for evaluating the performance of the technique.
Dextran	A complex branched glucan (polysaccharide) commercially available in diverse sizes, usually labeled with fluorescence. In intracellular delivery, dextran is extensively used as a characterization cargo, identifying the intracellular delivery performance of a method.
Diffusion	The process by which molecules and small particles move from one location to another by random and thermally driven motion. For membrane disruption‐mediated methods, diffusive transport facilitates small cargo delivery but is limited in transporting macromolecules.
Endocytosis	The cellular process in which proteins and other soluble small molecules in the extracellular milieu are internalized by being engulfed with a segment of the plasma membrane. In the intracellular delivery assay, cells subjected to endocytosis are often considered a negative control group.
Immortalized cell line	The population of cells derived from a plant or animal (human) capable of dividing indefinitely in culture.
Messenger RNA (mRNA)	RNA that specifies the order of amino acids in a protein (i.e., the primary structure). In intracellular delivery, mRNA is widely used for transient transfection.
Microfluidics/nanofluidics	Manipulation, control of fluids, or study of flow behaviors that are confined within micro/nanometer‐scale channels.
Plasmid DNA (pDNA)	A circular double‐stranded DNA molecule that can be replicated independently and used extensively as a vector carrying specific genes for cellular engineering by transformation. In intracellular delivery, a high level of delivery of plasmid DNA is regarded as one of the challenging tasks.
Primary cell	A cell isolated directly from plant or animal (human) tissue with limited growth potential in culture. Most primary cells are known to be more challenging to transfect compared with immortalized cell lines, regardless of the delivery method of choice.
Reynolds number (*Re*)	A nondimensional number given by *Re* = *ρuL*_c_/*η*, where *u* is the mean velocity of the flow, *L*_c_ is the characteristic length, *ρ* is the fluid density, and *η* is the fluid dynamic viscosity. The physical representation of the Reynolds number is that it is a measure of the ratio between inertial forces and viscous forces in a particular flow. In microfluidics and nanofluidics, the associated Reynolds number reaches close to zero due to small scale and low flow velocity, resulting in linear and predictable Stokes flow.
Transfection	The process of delivering nucleic acids (e.g., DNA or RNA) into eukaryotic cells for modulation of gene expression.

Given that this review focuses on microfluidically enabled (and nanofluidically enabled) intracellular delivery strategies, we will provide a detailed overview of the state‐of‐the‐art and recent advances in micro‐ and nanofluidic intracellular delivery methods. Acknowledging that there are reviews on conventional intracellular delivery methods,^[^ ^12^, ^13^, ^18^, ^19^, ^20^ ^]^ we will briefly reiterate them here. We will discuss the motivation behind the development of microfluidic and nanofluidic intracellular delivery approaches, their emergence as a new solution, and their synergistic consolidation with the general existing intracellular delivery methods, and comment on new opportunities for greater impact and breakthroughs. We will also elaborate on the limitations and challenges of current fluidically enabled solutions and share our perspectives on them. Finally, key considerations for future efforts to develop a micro‐ and nanofluidic device aimed at establishing a next‐generation intracellular delivery platform will be outlined.

## 2 Current Challenges of Intracellular Delivery and Motivations for Developing Micro‐ and Nanofluidic Solutions

**Figure 4:**
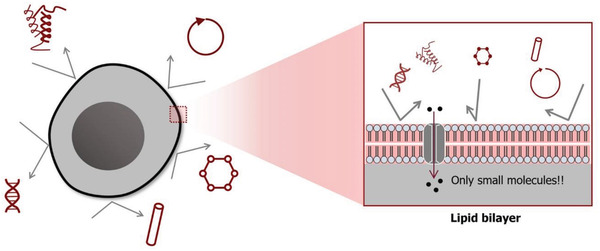
Schematic representation of a cell membrane. Selective permeability of the cell membrane, not allowing the entry of artificial and large cargos.

To internalize foreign biomolecules inside a living cell, external cargos must pass across the cellular membrane. The plasma membrane itself is a phospholipid bilayer consisting of two sheets of hydrophilic heads that face outward and nonpolar hydrophobic tails arranged tail‐to‐tail, as shown in **Figure** **4**. Because of the structural and chemical characteristics of phospholipids, only (lipophilic) small solutes and molecules can freely pass through the membrane. Furthermore, because the cellular membrane is negatively charged, cargos with the same polarity are naturally repelled. Therefore, the cell membrane selectively regulates the uptake of external cargos and acts as a semipermeable membrane, not allowing the entry of artificial and large cargos. It should be noted that this situation becomes even more complicated in the case of nucleic acid delivery. Cargos not only need to pass through the cell membrane, but also enter the nuclear envelope through nanoscale nuclear pores. Without proper chemical modification, passivation, or encapsulation, nucleases present in the cytosol quickly degrade naked nucleic acids before they can reach the nucleus.^[^ ^21^ ^]^

To circumvent these complications, carriers or membrane‐disruption modalities have been employed, as briefly mentioned above. Starting with carrier‐mediated methods, a carrier ferries cargo into cells as a transport vehicle. The carrier first encapsulates a cargo, and this encapsulation itself physically and chemically separates the cargo from the cellular environment (e.g., nuclease‐rich cytosol) to preserve the biostability of the cargo. Encapsulation compacts macromolecules (e.g., hundreds of nanometers plasmid DNAs) down to tens of nanometers in a spherical format, which facilitates large cargo delivery.^[^ ^22^ ^]^ Lipid, polymer, and cell‐penetrating peptides (CPPs) are nonviral carriers that take advantage of the cell's endocytic pathways to enter the cell.^[^ ^22^, ^23^, ^24^ ^]^ Cationic lipid is a representative nonviral carrier mainly designed for nucleic acid transfection, and Lipofectamine^®^, a commercialized product, is commonly used in the laboratory. Although the lipid‐based delivery approach offers relatively low cost and cytotoxicity in vitro and in vivo, lipid carrier methods suffer from endosomal entrapment (Figure 3a) and delayed unpacking, resulting in low and inconsistent delivery efficiency.^[^ ^25^, ^26^ ^]^ In particular, cationic lipid‐mediated delivery has shown limited efficiency in transfecting primary blood cell types^[^ ^27^ ^]^ because the internalization of lipid vesicles depends critically on interaction with the cellular membrane (i.e., cell type dependent). To detour these endocytosis‐associated barriers, direct fusion of carriers, such as cell ghosts and cell‐driven vehicles, has gained increasing attention.^[^ ^28^ ^]^ For example, exosomes are internalized through direct fusion; thus, an effective release of cargos into the cytosol is possible with minimal cell perturbation, demonstrating high potential as a new cargo carrier. However, isolation of exosomes involves a taxing isolation process, and loading cargos into exosomes is another critical hurdle.^[^ ^29^ ^]^ In summary, nonviral carrier approaches are limited owing to slow and inconsistent delivery, strong cell type dependence, long preparation steps, immunogenicity concerns, and low primary cell delivery efficiency.

To overcome these drawbacks, viral vectors have been extensively used for nuclear acid transfection.^[^ ^19^, ^30^ ^]^ Unlike nonviral carriers (except fusion‐based carriers), viral transduction takes advantage of the viral infection pathway, which can be free from endocytic complications. Although viral transduction is highly preferred because of its high cell uptake and transfection efficiency for diverse cell types, including primary cells, it must be noted that the design, optimization, and production processes are extremely laborious and costly, especially under cGMP regulations. Additionally, viral transduction results in high frequencies of off‐target events, oncogenicity, and adverse immune and inflammatory responses, indicating evident safety concerns.^[^ ^31^ ^]^ Furthermore, the limited payload capacity (cargo size) is considered another critical limitation in the versatility of this approach.^[^ ^32^ ^]^

Given this situation, potential microfluidic integration could offer new opportunities for carrier‐mediated intracellular delivery. Microfluidics can play definitive roles in carrier preparation, cargo encapsulation, chemical reaction control, reduction in reagent consumption, and/or automation. Nevertheless, the delivery efficiency after microfluidic integration with carrier‐based approaches is not anticipated to be taken to the next level because the delivery mechanism/principle remains the same. Moreover, carrier‐based strategies strongly depend on cell and cargo characteristics, implying that microfluidic design and/or operational conditions should be varied and optimized at times. Thus, instead of microfluidic integration with carrier‐mediated methods, the field has evolved to find methods and opportunities to synergistically consolidate microfluidics with the membrane disruption‐based methods discussed in the following sections.

## 3 Mechanical Plasma Membrane Disruption‐Mediated Intracellular Delivery (Mechanoporation)

**Figure 5:**
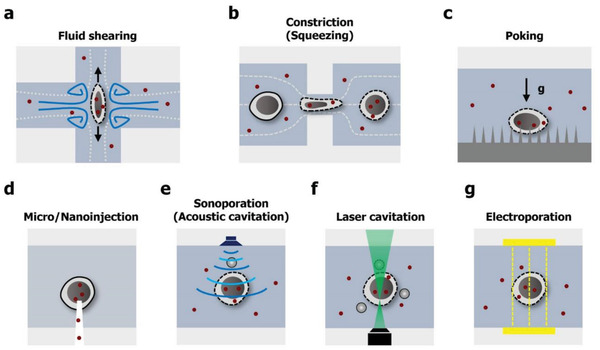
Microfluidic cell membrane disruption‐mediated techniques for intracellular delivery. Cell membrane deformation through a) fluid shearing, b) cell constriction, and c) nanoneedle penetration. d) Direct injection of external cargos through a hollow micro‐ and nanoconstruct. Cell membrane perforation via e) acoustic energy, f) optical, and g) electrical. Red dots indicate the target material.

In this section, we will introduce cell membrane disruption via mechanical (physical) means and subsequent intracellular delivery within microfluidic confinement, as shown in **Figure** **5**a–f. In this review, we begin by discussing the delivery mechanism and principle, provide a summary of currently developed delivery systems, and highlight new opportunities for cellular engineering research and applications through microfluidics and nanofluidics. In addition, we will discuss challenges of each approach and possibilities of further improvement.

### 3.1 Mechanisms of Mechanical Membrane Disruption

The major advantage of membrane disruption‐mediated intracellular delivery is that delivery is less sensitive to cell and cargo properties. The general principle of the membrane disruption‐based delivery approach is to transport cargos dispersed or suspended in solution into cells after physical membrane perforation via external forces. All micro(nano)fluidic techniques in this section have in common the fact that the plasma membrane is mechanically disrupted. Mechanical forces, through fluid shear, physical contact, microneedles, or acoustics, induce instability and consequently disrupt the lipid bilayer membrane, leading to cellular membrane disruption. Once the discontinuities (a.k.a. nanopores or nanoholes) on the cellular membrane are created, the nearby external molecules can be introduced into the cytoplasm through the created membrane nanopores by diffusion. The transport of cargos can also be guided actively, for instance, via microinjection, nanoinjection, and local convective flows.^[^ ^15^, ^33^, ^34^, ^35^ ^]^ The delivery of external cargo will last until the nanopores are resealed via membrane repair pathways utilizing tension reduction, exocytosis, and patch formation.^[^ ^36^, ^37^ ^]^ It is evident that cells exposed to excessive membrane perturbation fail to repair their membrane and exhibit apoptotic responses, resulting in cell death. Thus, it is critical to identify an appropriate mechanical cell perturbation method based on physical properties of the cell, such as cell size, deformability, and nuclear‐to‐cytoplasmic (NC) ratio, to yield maximum delivery efficiency while maintaining high cell viability.

### 3.2 Fluid Shear‐Induced Cell Deformation

Fluid shear‐induced cell deformation within a microchannel has been one of the major mechanical membrane disruption methods owing to its simple operational principle. Broadly speaking, fluidic shear induces torsion of the hydrophilic lipid of the cellular membrane, resulting in instability and rupture of the membrane bilayer.^[^ ^38^, ^39^ ^]^ Before discussing details of microfluidic intracellular delivery via fluid shear, we would like to highlight two important off‐chip studies on bulk fluid shear‐mediated intracellular delivery methods using a syringe and viscometer.

The first fluidic cell shearing concept for intracellular delivery was demonstrated using a conventional syringe.^[^ ^40^ ^]^ The cell and target molecule‐mixed suspension solution was prepared in a microtube and exposed to fluid shear stress by repeated infusion and withdrawal of the syringe piston using a microgauge needle. Consequently, fluid shear led to the permeabilization of the cellular membrane, allowing transport of foreign molecules into the cytoplasm. Using this extremely simple and cost‐effective approach, the delivery of 10 kDa fluorescein isothiocyanate (FITC)‐conjugated dextran, DNA,^[^ ^41^ ^]^ protein,^[^ ^42^ ^]^ and oligomer^[^ ^43^ ^]^ has been demonstrated. The syringe loading method pioneered fluid shear as an effective driving strategy of membrane disruption for intracellular delivery; however, the manual push and pull syringe piston operation is limited in terms of controllability and reproducibility of delivery performance. To address these drawbacks, a cell shearing device with a cone‐plate viscometer was developed.^[^ ^44^ ^]^ In contrast to a conventional parallel disk viscometer, the cone‐plate viscometer can generate uniform but tunable shear stress based on the radius of the cone.^[^ ^44^ ^]^ Thus, precise and uniform shear stress could be applied to the adhered cells below the viscometer, enabling delivery. Using the viscometer, Blackman et al. reported the uptake of 4 kDa dextran (16.4%) through mechanical disruption of adhered adult bovine aortic endothelial cells (ECs); however, low efficiency is considered a limitation.

A microfluidic system that allows consistent and robust molecule internalization in a high throughput manner has been explored, inspired by these two early studies of bulk, shear stress‐induced off‐chip intracellular delivery methods. In 2008, Hallow et al. reported one of the first microfluidic intracellular delivery systems employing fluid shear generated in narrow confinements.^[^ ^45^ ^]^ An array of microchannels was fabricated in cylindrical or conical shapes with diameters of 50–300 µm, using laser cutting of polyethylene terephthalate. The cell suspension containing calcein, FITC‐labeled dextran, or FITC‐labeled BSA as target molecules was injected through the microchannels via a syringe pump. Note that because the diameter of the channels is larger than that of cells, permeabilization of the cellular membrane is solely induced by high fluid shear. The delivery efficiency was characterized, and 36% of molecule uptake with 80% cell viability was reported. In particular, the introduction of macromolecules (2000 kDa FITC–dextran) was also demonstrated (uptake ≈10%), suggesting the possibility of large cargo delivery. Although this study shed light on microfluidic intracellular delivery via fluid shear, low uptake efficiency and unconventional microfluidic chip fabrication and design were indicated as limitations of the strategy.

In another approach, a microfluidic device attached to a conventional cell culture Petri dish was used for fluidically shearing neural cells.^[^ ^46^ ^]^ The microfluidic channel was fabricated using standard SU‐8 lithography and polydimethylsiloxane (PDMS) molding processes. Once the channel came in contact with the culture dish, the DNA‐loaded lipoplex solution was pumped through the channel using a syringe pump, and shear stress permeabilized the primary neuron cell membrane. Using this approach, transfection efficiencies of 9% and 44% were reported for primary neurons and neuron‐like N1E‐115 cells, respectively. However, these reported efficiencies were low, and a complicated fluid control system was used, thus lowering practicability.

**Figure 6:**
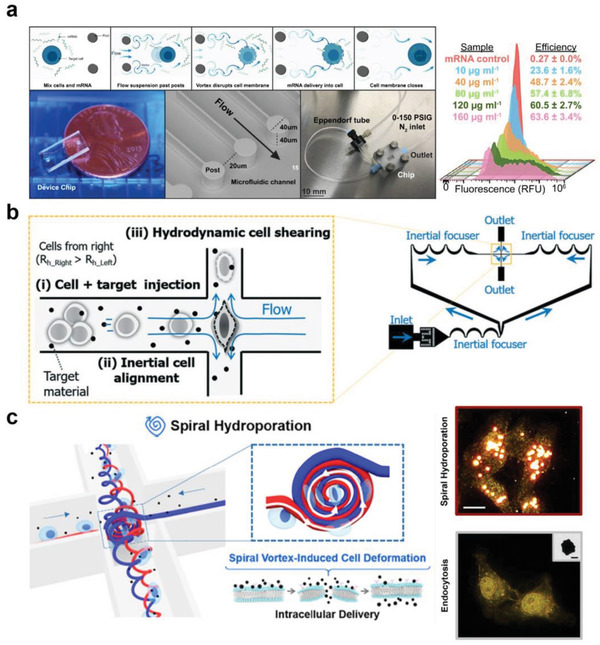
Fluid shear‐induced membrane permeabilization for intracellular delivery. Cell membrane disruption via a) vortex shedding, b) symmetric planar extensional flow, and c) spiral vortex and vortex breakdown. a) Reproduced with permission.^[^ ^48^ ^]^ Copyright 2019, Springer Nature. b) Reproduced with permission.^[^ ^49^ ^]^ Copyright 2019, Royal Society of Chemistry. c) Reproduced with permission.^[^ ^35^ ^]^ Copyright 2020, American Chemical Society.

Another strategy for generating fluid shear in microchannels is by employing vortex shedding, a well‐known flow oscillation motion behind a bluff body.^[^ ^47^ ^]^ Recently, Jarrell et al. reported a microfluidic vortex shedding device for transfecting human primary T lymphocytes (**Figure** **6**a).^[^ ^48^ ^]^ When the fluid passed the cylinders in the microfluidic channels, fluctuating vortices were generated behind the cylindrical structures. The induced vortices disrupted the lipid membrane of the cells, allowing the entry of external molecules. To optimize transfection performance, several conditions with different Reynolds numbers, reagent concentrations, and population of post array were explored, and a maximum of 64% of mRNA transfection efficiency of T cells was demonstrated. Notably, 43% of transfection yield, defined by multiplying transfection efficiency, viability, and recovery rate, was reported, and a large number of cells could be processed (≈2 × 10^6^ cells min^−1^) without significant channel clogging. Moreover, the stability of T cells was investigated, and the cells remained unaffected based on the evaluation of CD69 and CD25 marker expression levels. A decent transfection efficiency was achieved; however, an extremely high concentration of mRNA (160 µg mL^−1^) was used. Furthermore, the system is not ideal for processing small volume samples, and the applicability of plasmid DNA delivery has not yet been explored.

Recently, Kizer et al. presented a crossjunction microfluidic channel platform called Hydroporator for perforating the cell membrane via fluid cell shearing (Figure 6b).^[^ ^49^ ^]^ The cell suspension mixed with the target materials was injected into a cross‐slot microchannel at moderate Reynolds numbers. Inertial effects in microchannels^[^ ^50^ ^]^ were utilized to exert robust cell deformation at the stagnation point. The extensional flow stretched the cells and created membrane discontinuities, allowing rapid transport of external nanomaterials into the cells. The system achieved nearly 90% delivery efficiency for 3–5 kDa FITC–dextran delivery into K562 cells, and, importantly, the relationship between the delivery efficiency and intrinsic mechanical properties of the cells (i.e., deformability) was investigated. Plasmid DNA (pMAXClonning; 2.9 kbp) and DNA nanostructures were delivered into HEK293 and K562 cells, respectively. Approximately 32% of plasmid DNA transfection was achieved, presenting the possibility of macromolecule internalization. Note that the platform is free from channel clogging, which is one of the major drawbacks of microfluidics‐based approaches (more details can be found in Section 3.3.1). In a follow‐up study using a similar channel layout, an instability‐induced spiral vortex was utilized for intracellular delivery (Figure 6c).^[^ ^35^ ^]^ Using this vortex‐based cell deformation, extremely large nanoparticles (200 nm gold nanoparticles) were successfully delivered into cells. Although these studies have demonstrated the capability of internalizing diverse nanomaterials into cells via fluid shear stress, large size plasmid DNA delivery has not been investigated.

### 3.3 Physical Contact

Mechanical cell membrane disruption has also been explored by physically interfacing cells with solid structures within microchannels. This method includes two strategies: 1) passing cells through a series of narrow constriction channels that have a smaller width than the diameter of the cells, creating membrane discontinuities, and 2) poking cells with a sharp channel structure(s) to perforate the lipid bilayer.

#### 3.3.1 Constriction

Off‐chip internalization of external molecules into cells using constrictions was first reported in 1999 as a proof‐of‐concept study known as “filtroporation.”^[^ ^51^ ^]^ Polycarbonate microporous membranes were manually mounted on a filter holder connected to a collection tube underneath. Suspended Chinese hamster ovary (CHO) cells were mixed with fluorescein‐labeled dextran of different weights (10, 70, and 500 kDa) or luciferase reporter plasmid vectors (5.3 kbp). A pneumatic source was used to introduce the sample to force the cells to pass through the micropores. By testing various diameters of micropores (5–20 µm), the micropore with a diameter smaller than that of the cell yielded significantly increased uptake of 10 kDa dextran, up to 60%, with cell viability of 70%. Cell transfection with luciferase encoding plasmid DNA, using an 8 µm micropore membrane, was also demonstrated (no quantitative data on delivery efficiency was reported). Extending this concept, a scale‐up system using a 24‐membrane manifold system was reported.^[^ ^52^ ^]^ Based on this study, 63.1% of *β*2‐microglobulin (B2M)‐knockout efficiency in human hematopoietic stem and progenitor cells (HSPCs) was achieved with Cas9 ribonucleoprotein (RNP)‐based delivery, demonstrating primary cell applicability. However, system complexity and inconsistency in delivery and viability are considered drawbacks of the platform.

**Figure 7:**
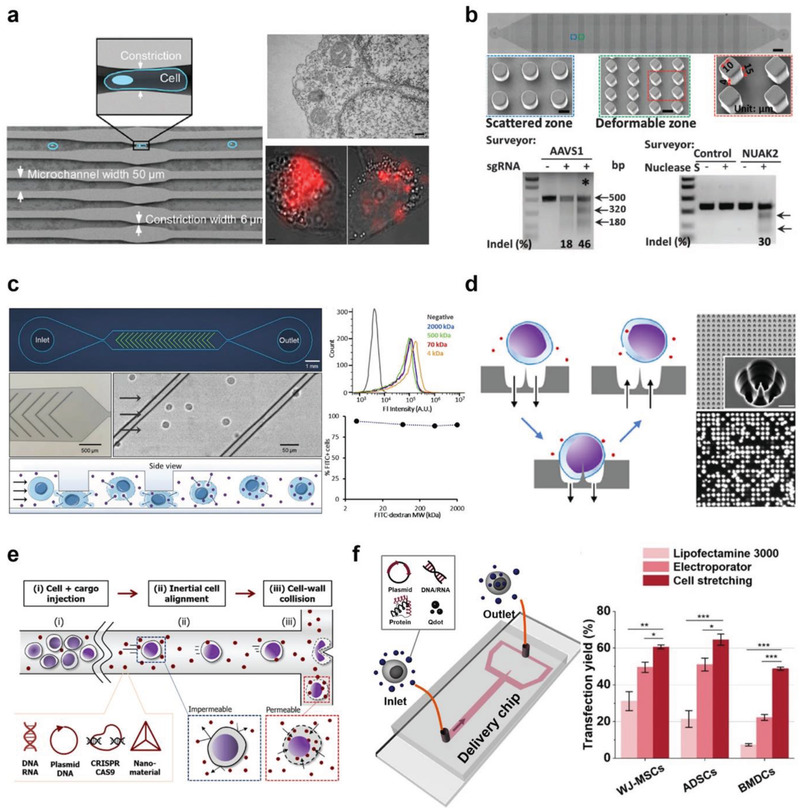
Microfluidic cell mechanoporation strategies through physical contact between cells and channel geometry. Cell membrane disruption by passing cells through a) narrow constrictions (also called cell squeezing), b) a microconstriction array, and c) vertical ridges. Cell membrane perforation using d) a nanoneedle penetration, and e) cell poking through fluid inertia. f) Hydrodynamic cell stretching induced intracellular delivery. a) Reproduced with permission.^[^ ^53^ ^]^ Copyright 2013, National Academy of Sciences. b) Reproduced with permission.^[^ ^58^ ^]^ Copyright 2015, American Association for the Advancement of Science. c) Reproduced with permission.^[^ ^33^ ^]^ Copyright 2018, Elsevier. d) Reproduced with permission.^[^ ^77^ ^]^ Copyright 2020, American Chemical Society. e) Reproduced with permission.^[^ ^78^ ^]^ [Copyright 2018, American Chemical Society. f) Reproduced with permission.^[^ ^15^ ^]^ Copyright 2020, American Chemical Society.

Toward higher controllability and consistent delivery, new efforts have led to the introduction of microfluidics. This is largely because the microfluidic platform provides a set of knobs to tune parameters, such as constriction dimension and flow condition, simply by modulating the channel geometry or pressure (i.e., flow rate) to identify the optimized delivery condition. Jensen and Langer groups pioneered a microfluidic intracellular delivery approach using a series of constrictions within microchannels, where the approach was named “cell squeezing” (**Figure** **7**a).^[^ ^53^, ^54^, ^55^ ^]^ An etched silicon microchannel containing bottlenecks with widths of 4–8 µm and constriction lengths of 10–40 µm was used. The cells were injected into microchannels by a pneumatic setup, forcing cells to pass through the constrictions. Since the constriction width was designed to be half of the cell diameter, the cells experienced substantial membrane disruption, enabling the transport of external molecules into the cytoplasm through the created discontinuities. The study substantiated that repeated constriction could increase the delivery efficiency of 3 kDa FITC–dextran in HeLa cells to ≈75%.^[^ ^53^ ^]^ Furthermore, a wide range of cell types, including primary cell lines (e.g., primary fibroblasts, dendritic cells, blood immune cells, and embryonic stem cells (ESCs)), were processed with various nanomaterials, such as dextran, siRNA, carbon nanotubes, gold nanoparticles, and transcription factors. Despite the delivery of diverse macromolecules into different cell types, it should be noted that the major limitation of this strategy is the inability of plasmid DNA transfection.

To address this challenge, an electric field was combined with the previous cell squeezing method to enable active transport of DNA into the nucleus.^[^ ^56^ ^]^ A set of electrodes was added after a single constriction for sequential physical and electrical cell perturbations (details of electroporation‐based intracellular delivery are described in Section 4). Using this hybrid system, green fluorescent protein (GFP)‐plasmid DNA (size not reported) transfection of HeLa cells was demonstrated with 60–90% transfection efficiency. Additionally, more than 80% of GFP expression occurred in the first hour after treatment, which is significantly faster than that observed with conventional electroporation (4–48 h after treatment). The main claim in this study is that physical disruption of the membrane by cell squeezing opens the membrane, and active electrophoretic transport of DNA is achieved by an external electrical field similar to electroporation, which accounts for rapid DNA delivery and transfection. However, the complexity of the additional electrical components and device fabrication critically lowers its practicability.

Recently, a subsequent study demonstrated the transfection of primary human T cells with Cas9‐gRNA RNP targeting PD‐1 for cancer immunotherapy applications.^[^ ^57^ ^]^ The same cell squeezing method was used, attaining 46.7% editing efficiency of human T lymphocytes similar to that of electroporation (50.3% using 4D‐Nucleofector System, Lonza). Although both methods showed comparable delivery efficiency of editing primary T cells to that of electroporation, the electroporated T cells exhibited severe and undesired upregulation of IL‐2 (648‐fold), IL‐9 (33‐fold), IFN‐*γ* (30‐fold), and TNF‐*α* (10‐fold), which was not observed in cells processed via cell squeezing. Furthermore, electroporated T cells misexpressed more genes than cells processed via cell squeezing (34% vs 9% of all genes). The authors hypothesized that these disparities would cause differences in in vivo therapeutic efficacy and validated this by confirming effective tumor size reduction from PD‐1‐edited T cells via cell squeezing. Note that this study clearly demonstrates the fundamental benefits of mechanoporation over electroporation; however, transfection level could not surpass that of electroporation, and transfection was performed at a high concentration (100 µg mL^−1^) of RNP. In addition, potential channel clogging due to bottleneck design would cause operational failure.

Instead of an array of single straight channels with constrictions, the Qin group used a microchannel with a constriction/obstacle array to achieve intracellular delivery via cell constriction (Figure 7b).^[^ ^58^ ^]^ Although the design can mitigate the clogging issue, the intracellular delivery principle is the same. The PDMS–glass microfluidic device layout is a cost‐effective solution compared with the silicon‐based cell squeezing method discussed above. The microchannel comprises 10 arrays of diamond‐shaped microstructures with 4 µm gaps for repeated mechanical deformation of cells as they pass through the constriction array. Using the presented approach, 80% of ssDNA delivery efficiency was reported for HEK293t cells, and the Akt1 gene in PC‐3 cells was knocked down by internalizing siRNA with 70% efficiency. Furthermore, decent transfection efficiency (30–60%) of the human lymphoma cell line (SU‐DHL‐1) and mouse embryonic stem cell line (AB2.2) with plasmid DNA encoding enhanced green fluorescent protein (EGFP) was reported; however, key information on DNA concentration and plasmid DNA size was not reported. The same group further modified the constriction shape from diamond‐shaped to star‐^[^ ^59^ ^]^ and branch‐shaped^[^ ^60^ ^]^ arrays for CRISPR‐Cas9 RNP delivery (>40% indel efficiency) and siRNA internalization, respectively.

Recently, Liu et al. reported a different microchannel layout for mechanical cell deformation‐induced biomolecule delivery (Figure 7c).^[^ ^33^ ^]^ Instead of horizontal constrictions from cell squeezing, the approach utilizes vertical ridges. Transient cell volume exchange induced cargo delivery was demonstrated by passing cells over ridges. Although the deformation approach shares some features with the cell squeezing approach (i.e., passing cells through narrow constrictions), the authors claimed that their delivery was purely based on convective transport, whereas diffusion was the only delivery mechanism for cell squeezing. Harnessing the platform, more than 90% of dextran‐delivered K562 cells was reported, regardless of dextran sizes (4–2000 kDa) (Figure 7c). Moreover, this study achieved 67% mRNA transfection efficiency of K562 cells and showed the possibility of plasmid DNA (5.8 kbp) transfection (43%). The platform also demonstrated the applicability of transfecting primary peripheral blood mononuclear cells (PBMCs) with mRNA (38%). However, a relatively low primary cell transfection efficiency has been reported, using high concentrations of nucleic acid reagents. As a follow‐up study, the same group investigated cell integrity after the device process by assessing the stability of the nuclear envelope and intracellular contents.^[^ ^34^ ^]^ To analyze nuclear envelope integrity, HEK293 cells were transformed with two reporter vectors to evaluate nuclear envelope rupture and the loss of nuclear contents. After delivery, nuclear membrane damage was confirmed using fluorescence imaging, but the loss of nuclear contents was claimed to be insignificant. In contrast, the cells subjected to electroporation showed both nuclear damage and loss of nuclear contents, suggesting that microfluidic mechanoporation was less invasive than electroporation, although no cell functional studies have been reported.

Most microfluidic constriction‐based intracellular delivery approaches^[^ ^33^, ^53^, ^58^, ^61^, ^62^, ^63^, ^64^ ^]^ provide high scalability, simplicity of operation, and cell type insensitive delivery with decent delivery efficiency. However, drawbacks such as channel clogging, inconsistent delivery due to cell size heterogeneity, low plasmid DNA transfection efficiency, and large cargo consumption should be addressed for wider use as a next‐generation intracellular delivery method.

#### 3.3.2 Poking Cells to Perforate the Membrane

Advances in nanotechnology have allowed the fabrication of a wide range of nanostructures, including nanoneedles, nanowires, and nanostraws.^[^ ^65^ ^]^ These sharp nanostructures have been found to pierce the cell membrane and nuclear envelope, creating transient nanopores that enable intracellular delivery.^[^ ^66^ ^]^ There are excellent review articles on intracellular delivery via purely nanostructure‐based cell penetration.^[^ ^67^, ^68^ ^]^ Thus, we will focus on discussing how nanostructures can be synergistically integrated with microfluidics. Note that intracellular delivery with micro‐ and nanoinjections (cargo delivery through a hollow micro‐ and nanoconstruct) are separately discussed in Section 3.4 and “Nanochannel electroporation,” “Nanostraw electroporation,” and “Nanofountain electroporation” sections, respectively.

Starting with off‐chip devices, the first intracellular delivery through direct penetration with nanoneedles was demonstrated on a bulk scale in early 2000.^[^ ^66^, ^69^ ^]^ For example, in 2007, Kim et al. reported the intracellular delivery of DNA using a silicon nanowire array.^[^ ^70^ ^]^ For nucleic transfection, plasmid DNA was deposited on the tips of nanowires before the cells were cultured on top of the nanowire array. Using confocal microscopy, it was observed that the nanowires penetrated the cell membrane, generating discontinuities for delivery. Although successful plasmid DNA transfection was observed through fluorescence microscopy, cell culturing above the nanowire array revealed several concerns, such as decreased cell viability in long‐term proliferation and cell dysfunction due to DNA damage.^[^ ^71^, ^72^ ^]^ Furthermore, it was recently reported that nanostructures with specific geometries fail to permeabilize the cellular membrane because the nanostructure can conform to the cell membrane without spontaneously rupturing,^[^ ^73^ ^]^ yielding no intracellular delivery. Therefore, for effective cell membrane permeabilization, an external driving force is generally used to enhance membrane perforation. For instance, Wang et al. utilized a standard centrifuge to pierce the adhered cells with a diamond nanoneedle array, generating nanopores on their lipid membrane.^[^ ^74^ ^]^ Briefly, mechanical penetration by a nanoneedle was made by centrifugal force to transfect primary neuron cells with GFP plasmid DNA–lipid complexes, demonstrating a transfection efficiency of 45%. However, this method is difficult to use with suspension cell types and involves costly and complex fabrication procedures.

To address these challenges, such as low controllability of cell penetration, inapplicability to suspension cells, low scalability, and complex fabrication of nanoneedle‐based intracellular delivery, microfluidic approaches have been investigated. Micro/nanotechnologies have allowed the facile fabrication of sharp features on the channel surface, such as tips, protrusions, and blade shapes, and the cell suspension can be injected into fluidic channels containing these sharp nanostructures. Furthermore, the cell suspension injected into the microchannels can be processed in a continuous manner, allowing high scalability. For example, Ma et al. reported CRISPR‐Cas9 delivery by poking cells using spiky microchannel surfaces called nanoblades.^[^ ^75^ ^]^ A silicon mold was fabricated using standard photolithography and reactive ion etching (RIE) to create 200 nm radius nanoblades. Hematopoietic stem cells (HSCs) were injected into a microchannel with nanoblades at 50 µL min^−1^, and the cells were mechanically disrupted, leading to transient permeabilization of the cell membrane. As a result, 70% delivery efficiency of 70 kDa dextran into HSCs with 80% viability was demonstrated, and successful delivery of C/EBP*α* targeting Cas9 RNP was also presented. Another study by Xing et al. employed two localized point sharp geometries termed “point constrictions” in microchannels to breach the cell membrane.^[^ ^76^ ^]^ The microfluidic device was prepared by etching a silicon wafer bonded to a Pyrex wafer. The cells mixed with dextran or siRNA were pumped at constant pressure using a pneumatic setup. Diverse mammalian cell types, including NIH3T3, HEK293, MDCK, and HCT116, were showcased the delivery of 3 and 70 kDa FITC–dextran. The platform achieved 65% antitubulin antibody delivered HCT116 cells and 60% gene knockdown efficiency of siRNA with HeLa cells. However, as described by the authors, the system failed to achieve plasmid DNA transfection similar to that observed with cell squeezing. Moreover, the fabrication process is labor‐intensive, and the channel layout inevitably carries the risk of channel clogging.

As an alternative, Dixit et al. presented a parallelized single‐cell penetrator for intracellular delivery (Figure 7d).^[^ ^77^ ^]^ An array of single‐cell penetrators was fabricated by sequentially etching silicon on an insulator substrate, creating a total of 10^4^ cell penetration sites. Each penetrator site had aspiration vias for capturing cells by applying a negative pressure and for penetrating the cells using a nanoneedle located in the penetrator site center. It is believed that external cargos dispersed in the suspension diffused into the cytoplasm after cells were released from the penetrator sites. Processing with the system, Jurkat, K562, and primary human T cells were transfected with GFP plasmid DNA (4.7 kbp), and the results were compared with those of cells treated with a commercial bulk cuvette‐based electroporator (Nucleofector, Lonza). The authors reported that the platform showed 88%, 49%, and 82% of transfection yield for Jurkat, K562, and primary T cells, respectively, which were higher than those obtained using electroporation. The study claimed that the viability of processed cells reached nearly 100%, showing no significant difference when compared with that of the control group. However, the system involves an extremely labor‐intensive and time‐consuming device fabrication process, as well as a complex operational process. Furthermore, the low‐throughput feature due to the limited capture rate (≈71%) is another hurdle for application in cell‐based therapy.

Recently, the Chung group introduced a novel microfluidic intracellular delivery platform called inertial microfluidic cell hydroporator (iMCH) (Figure 7e).^[^ ^78^ ^]^ Through collision (i.e., poking) of cells with a sharp tip located at the T‐junction stagnation point of the microchannel, transient cellular membrane discontinuities were created, permitting the introduction of external macromolecules. The microfluidic chip on a 3 in. × 1 in. glass slide was prepared using a standard SU‐8 master for PDMS replication. The cell suspension was injected at a moderate Reynolds number, which is a key distinction between this and other techniques, taking advantage of the inertial effects presented in microfluidics^[^ ^50^ ^]^ for precise cell positioning and high cell processing rate (10^6^ cells min^−1^). By poking cells with a sharp tip at a high flow rate, successful delivery of various target molecules, such as 3 kDa FITC–dextran (>85%), siRNA, CRIPSR‐Cas9, plasmid DNA (≈45%), and different shapes of DNA origami nanoconstructs (54%), was demonstrated, while maintaining cell viability of more than 75%. Although this method presented highly effective and robust intracellular delivery with a cost‐effective and simple cell processing procedure, the potential clogging issue also applies to this platform.

To develop a platform with near‐zero risk of channel clogging and higher delivery performance, the same group reported a unique T‐junction microchannel with a microcavity structure (Figure 7f).^[^ ^15^ ^]^ The cavity structure was introduced to exert recirculating flows developed in the T‐junction at moderate Reynolds numbers, which substantially mitigates channel clogging. Since cell mechanoporation was conducted by sequential physical cell‐wall collision and fluid shearing via recirculating vortices, highly efficient delivery of diverse nanomaterials (e.g., 2000 kDa FITC–dextran, mRNA, siRNA, 7.9 kbp plasmid DNA, and 300 nm nanoparticles) into various cell types, including clinical primary cells, was achieved. Among them, highly effective plasmid DNA transfection (80%) of HEK293t cells was attained without the aid of a carrier or electric field. Furthermore, the platform exhibited superior mRNA transfection yield of hard‐to‐transfect primary stem and immune cells (i.e., human mesenchymal stem cells, adipose‐derived stem cells, and murine dendritic cells) compared with traditional benchtop techniques (i.e., Lipofectamine 3000 and capillary electroporation; Neon Transfection System, Invitrogen), showing high potential for cell‐based therapeutic applications.

### 3.4 Microinjection

#### 3.4.1 Conventional Microinjection

Traditional microinjection has been one of the most popular benchtop techniques for delivering a wide range of nanomaterials, including antibodies,^[^ ^79^ ^]^ quantum dots,^[^ ^80^ ^]^ and purified DNA,^[^ ^81^ ^]^ into various cell types. Microinjection was first invented in 1911 by Barber, using microdiameter glass pipettes.^[^ ^82^ ^]^ In brief, a hollow micropipette directly pierced the cellular membrane, and the solution with external cargos was injected through the pipette using pressure‐driven or electrokinetic flow.^[^ ^83^ ^]^ An additional pipet was often used with an independent pressure source to capture and position the target cell.^[^ ^82^ ^]^ Since the sharp pipette directly penetrated the cellular membrane and external cargos were injected through the needle, the approach is not restricted to cell and cargo types. However, only a trained/experienced user is able to perform microinjection, limiting its applicability, and the throughput of the system is extremely low, making it difficult to deal with a large cell population (skilled personnel can process ≈100 cells h^−1^).^[^ ^84^ ^]^ Furthermore, it is well documented that microneedle penetration often causes considerable cell damage, especially in small cell types.^[^ ^85^, ^86^ ^]^ The high cost of the system is another major factor limiting its widespread adoption in the field. To address these limitations, on‐chip microfluidic and nanofluidic injections have been proposed. Please refer to other literature for off‐chip nanoinjection,^[^ ^87^, ^88^ ^]^ and here, we will discuss how microfluidics and nanofluidics have been integrated for direct injection of external cargos.

#### 3.4.2 Microfluidic Microinjection

**Figure 8:**
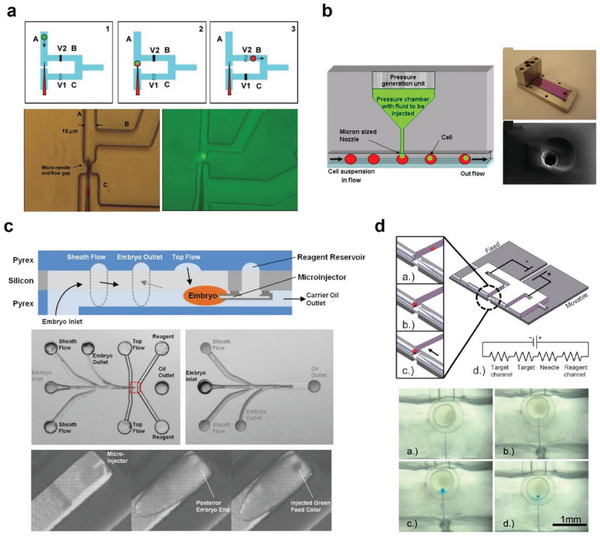
Microfluidic microinjection techniques. a) Single‐cell trapping and injection within a microfluidic confinement. Reproduced with permission.^[^ ^84^ ^]^ Copyright 2008, Royal Society of Chemistry. b) Single cell‐based cargo jet injection into flowing cells. Reproduced with permission.^[^ ^90^ ^]^ Copyright 2013, IOP Publishing. c) On chip *Drosophila* embryo microinjection system and microchannel design. Reproduced with permission.^[^ ^91^ ^]^ Copyright 2012, Royal Society of Chemistry. d) Electroosmotic methylene blue microinjection into zebrafish embryos. Reproduced with permission.^[^ ^83^ ^]^ Copyright 2009, Royal Society of Chemistry.

As an initial effort, Lee et al. introduced the concept of on‐chip microinjection.^[^ ^89^ ^]^ A glass microneedle fabricated by a micropipette puller was inserted into a PDMS‐based microchannel. The PDMS‐based microvalve was used to precisely dispense a small volume of fluid (less than 1 nL), but no intracellular delivery was demonstrated. Thereafter, Adamo and Jensen presented a microfluidic single‐cell injection system,^[^ ^84^ ^]^ employing a pulled glass microneedle embedded in a PDMS microchannel that can be controlled by a 3D microstage under a microscope (**Figure** **8**a). The single cell was first positioned in the injection area and pierced by the microneedle using pneumatic valve enabled flow control. The external molecules were then injected using a syringe pump.^[^ ^84^ ^]^ As a proof‐of‐concept, suspended HeLa cells were introduced into the microchannel for 10 kDa tetramethylrhodamine–dextran injection. Using this approach, a throughput of 3600 cells h^−1^ was reported, which was a great improvement over that of the conventional microinjection. However, the platform suffers from channel clogging issues. In a follow‐up study, the same group reported a microfluidic delivery platform by jetting droplets containing cargos into flowing cells (Figure 8b).^[^ ^90^ ^]^ A micronozzle was connected to the microchannel, and a sub‐picoliter (pL) volume of the fluid jet containing the target molecules was dispensed into cells by a piezoelectric actuator once the cells passed the micronozzle region.^[^ ^90^ ^]^ The continuous delivery of 10 kDa fluorescence‐labeled dextran into HeLa cells was demonstrated. However, the system complexity in operation and fabrication lowers the practicability, and asynchronization between cargo injection and cell positioning results in inconsistent delivery.

Along with on‐chip microfluidic microinjection efforts, automating the injection process has also been attempted. Delubac et al. reported an automated injection system for *Drosophila* embryos using a microchannel integrated with a microinjector (Figure 8c).^[^ ^91^ ^]^ The microinjector was fabricated by DRIE of a silicon wafer and anodically bonded to Pyrex, creating a Pyrex–silicon–Pyrex sandwich microfluidic chip. *Drosophila* embryos were introduced into the microchannels using a syringe pump and aligned with the sheath fluid. When an embryo was detected, 100 pL of reagent was injected into the embryo processing at ≈17 embryos min^−1^. By harnessing the platform, 87% of the embryos transfected with siRNA against EGFP exhibited silenced or reduced fluorescence signals. Although this system showed promise in the automation of microinjection, only partial automation was demonstrated, and the system complexity from fabrication and operation reduced its feasibility.

Another on‐chip microinjection was designed for precise nucleic acid transfection application. In 2009, Noori et al. reported on a microfluidic microinjector using electroosmosis as the driving force for injection (Figure 8d).^[^ ^83^ ^]^ The PDMS microchannel was bonded onto a glass slide, and a suction capillary, an injection needle, tubes, and electrodes were inserted into the microfluidic platform. The cells were first immobilized by a suction capillary, and the needle was positioned to pierce the cells by injecting an external reagent via electroosmotic flow. As a proof‐of‐concept, methylene blue dye was injected into zebrafish embryos, and its internalization was observed under a microscope. Since this platform employed electroosmotic flow, decent controllability of the delivered reagent dosage was demonstrated. However, the platform had several drawbacks, including clogging, needle fracture, and a time‐ and labor‐consuming alignment process during fabrication.

Although microfluidic integration with microinjection has allowed improved throughput,^[^ ^84^, ^92^ ^]^ dosage control,^[^ ^83^, ^93^ ^]^ automation,^[^ ^91^ ^]^ and usability,^[^ ^94^ ^]^ most are limited in system complexity, low scalability, reproducibility, and inconsistent delivery, and these should be addressed to enable wider usage and applications.

### 3.5 Cavitation

**Figure 9:**
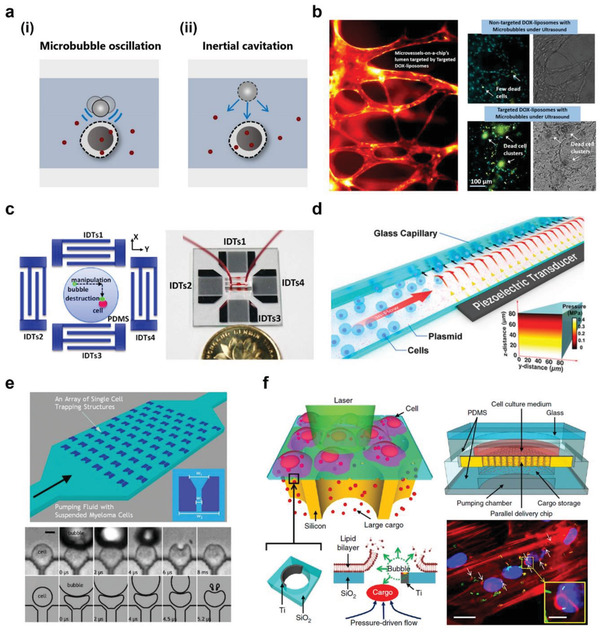
Microfluidic sonoporation and cavitation for intracellular delivery. a) Schematics of membrane disruption using i) microbubble oscillation and ii) inertial cavitation. b) Intracellular delivery into microvessels using microbubble oscillation. Reproduced with permission.^[^ ^114^ ^]^ Copyright 2016, American Chemical Society. c) Surface acoustic wave (SAW) microbubble destruction induced cell permeabilization. Reproduced with permission.^[^ ^115^ ^]^ Copyright 2014, American Institute of Physics. d) Acoustofluidic sonoporation (a combination of acoustic pressure, shear force, and cavitation) for cell membrane discontinuity. Reproduced with permission.^[^ ^117^ ^]^ Copyright 2020, National Academy of Sciences. e) Microfluidic array for parallelized single‐cell intracellular delivery via inertial cavitation. Reproduced with permission.^[^ ^119^ ^]^ Copyright 2013, Royal Society of Chemistry. f) Laser‐induced cavitation for macromolecules intracellular delivery. Reproduced with permission.^[^ ^120^ ^]^ Copyright 2015, Springer Nature.

In the 1980s, cavitation was proposed for creating cell membrane discontinuities as an alternative to electroporation or viral vectors for gene transfection.^[^ ^95^ ^]^ Microscale bubbles can be generated and/or manipulated by external sources such as ultrasound^[^ ^96^ ^]^ and lasers.^[^ ^97^, ^98^ ^]^ The sudden deposition of energy into the fluid leads to the creation of cavitation bubbles, and the generated microscale bubbles can be controlled by modulating ultrasound waves or laser pulses. As shown in **Figure** **9**a, there are two major strategies for applying shear stress to adjacent cells, permeabilizing the cell membrane: oscillation of microbubbles, and drastic expansion and destruction of microbubbles (also called inertial cavitation). For example, low acoustic pressure or modulation of the pulse width of a laser can stably oscillate microbubbles, thus disrupting the cellular lipid bilayer^[^ ^99^, ^100^ ^]^ and transiently opening the cellular membrane to permit internalization of external materials. Regarding inertial cavitation, high acoustic pressure or laser energy can expand and collapse a microbubble, inducing fluid flow to fill the void. This cavitation phenomenon is adopted to create membrane discontinuities by imposing shear force on the cells.^[^ ^98^, ^101^ ^]^ In the following sections, we will briefly discuss acoustic‐ and laser‐assisted cavitation techniques and describe how they have been integrated with microfluidics for advanced intracellular delivery.

#### 3.5.1 Sonoporation and Cavitation

Acoustofluidic technologies have contributed to the development of innovative approaches for not only cellular analysis^[^ ^102^ ^]^ but also for intracellular delivery, termed sonoporation. The simplest macroscale sonoporation method, without microbubbles, utilizes a traditional sonicator for molecular delivery. Several mammalian cells, including HeLa, mouse myeloma, LTK‐fibroblast, and REF, were tested by sonication, and a maximum of 20% of 40 kDa dextran was introduced into fibroblasts.^[^ ^95^ ^]^ In addition, plasmid DNA was internalized into LTK fibroblasts and REF cells by sonication, and transfection was confirmed by imaging the transformed cell colonies. Although the approach is cost‐effective and simple to operate, low delivery and transfection efficiency are the major limitations.

To achieve a comparatively higher transfection efficiency, the introduction of bubbles and contrast agents into the solution was proposed for off‐chip sonoporation strategies.^[^ ^103^ ^]^ It is believed that ultrasound contrast agents facilitate the formation of stable microbubbles, increasing shear stress on cells upon microbubble destruction.^[^ ^104^ ^]^ Greenleaf et al. reported that a contrast agent assisted the sonoporation of immortalized human chondrocytes by locating an ultrasound transducer below a 6‐well plate cultured with cells.^[^ ^103^ ^]^ Approximately 43% of GFP plasmid DNA transfection (5 kbp) was achieved, and a 20‐fold delivery increase was demonstrated compared with that of a previous study.^[^ ^105^ ^]^ Despite such improvements, sonoporation on a macroscale still suffers from limited controllability of the acoustic wave pressure or generated microbubbles (e.g., number, location, and homogeneity), leading to low efficiency and inconsistent delivery.^[^ ^106^ ^]^ Moreover, because of difficulties in manipulating the location of the bubbles, the cavitation effect occurred randomly, causing excessive local shear stress on the cells, which resulted in high cell death.^[^ ^106^ ^]^ Several alternative studies employed acoustic pulses or waves without microbubbles (acoustofection) to deliver external materials, including propidium iodide (PI) dye, siRNA, and gold nanoparticles (AuNPs) (10 nm).^[^ ^107^, ^108^, ^109^, ^110^ ^]^ Although these techniques retained high cell viability after process since they permeabilize the membrane by lipid bilayer reorganization instead of poration, such platforms are limited in throughput and macromolecule delivery is unexplored. To overcome aforementioned challenges, sonoporation on microfluidic chips, with or without microbubbles, has been proposed. The overarching goal is to precisely control and manipulate the acoustic force or cavitation effect of microbubbles, enabling consistent and effective intracellular delivery of target nanomaterial.

As an initial microfluidic approach, an ultrasonic standing wave was used to control shear stress and migrate cells without microbubble generation.^[^ ^111^ ^]^ In this study, the purpose of cargo delivery was to induce cell death by introducing cytotoxic drugs into the cells. Ultrasonic waves at the resonant frequency generated by a piezoelectric transducer aligned and sheared cells for transient membrane permeabilization. Through the membrane pore, several therapeutic agents, such as doxorubicin, apigenin, and luteolin, were introduced into H9C2 cells, as confirmed by the increased cytotoxicity of doxorubicin up to 91%. However, this study only demonstrated small molecule delivery because the applied shear was insufficient to create large membrane pores for the delivery of macromolecules. Later, Dixon et al. proposed a sonoporator by generating microbubbles employing a flow‐focusing microfluidic device (FFMD)^[^ ^99^ ^]^ widely adopted for droplet microfluidics.^[^ ^112^ ^]^ Monodisperse microbubbles (droplets) formed by FFMD flowed over the primary rat muscle cells and were stably oscillated by an external ultrasound transducer. Microbubble oscillations induced shear stress on cells, creating cellular membrane discontinuities for the delivery of calcein into the cytoplasm (≈80%). Note that the study did not collapse the microbubbles but oscillated the monodisperse microbubbles stably enough to induce gentle shear stress for consistent molecule delivery. Although the study presented a novel sonoporation on‐chip technique, it is limited in its operational complexity and lack of functional material delivery.

A similar principle was applied beyond the single‐cell level, for instance, intracellular delivery into microvessels (Figure 9b). Note that microfluidic 3D cell culture has been a popular method for mimicking blood vessels or other tissue structures^[^ ^113^ ^]^ but the introduction of external molecules within a chip is problematic. Park et al. reported a method that could effectively deliver drugs (e.g., doxorubicin) into the microvessel on a chip by using microbubbles generated by off‐chip vial shaking.^[^ ^114^ ^]^ Ultrasound was then applied using a focused ultrasound transducer that stably oscillated microbubbles for the permeabilization of microvessels, and delivery was confirmed by liposomal doxorubicin internalization. Although the possibility of delivering drug material into a 3D cell culture microfluidic chip was exhibited, nontrivial microbubble generation setups and lipid carrier encapsulation steps were accompanied.

As an alternative, Meng et al. used a surface acoustic wave (SAW) for the sonoporation of a single cell (Figure 9c).^[^ ^115^ ^]^ The SAW device was fabricated by depositing an array of interdigital transducers (IDTs) on a piezoelectric substrate, as previously reported.^[^ ^116^ ^]^ The microbubbles generated from the off‐chip were injected into a cylindrical PDMS microchannel and positioned at the center of the IDTs via a syringe pump. Then, SAW was applied for the destruction of the microbubbles by a single‐shot pulsed radiofrequency signal. The precise control of microbubble cavitation transiently opened the cell membrane of MCF7 cells, facilitating the uptake of PI and fluorescein diacetate. While this study investigated the control of microbubbles and applied it for MCF7 dye intracellular delivery, no investigation of functional nanomaterial delivery to primary cell lines was conducted. Low throughput is an additional concern, making it difficult to use this technique for general intracellular delivery purposes.

Recently, Belling et al. introduced a bubble and contrast agent free acoustofluidic sonoporation system for intracellular delivery using a glass microcapillary that can process up to a throughput of 200 000 cells min^−1^ (Figure 9d).^[^ ^117^ ^]^ The system utilized the acoustic pressure waves generated by the piezoelectric (PZT) transducer to permeabilize the cell membrane and localize the flowing cells toward the DNA‐coated glass capillary wall. A combination of forces, including acoustic pressure, shear force, and cavitation, created membrane pores, allowing the introduction of exogenous biomolecules that were functionalized on the channel wall surfaces. Using the platform, 62% of EGFP plasmid DNA (4.5 kbp) transfection efficiency was achieved with 80% viability using Jurkat cells. Furthermore, primary cells, such as PBMCs and hematopoietic stem cells, were transfected as well, and an efficiency of up to 20% was reported for plasmid DNA transfection. The authors also investigated the possibility of nuclear membrane permeabilization by observing the nuclear localization signal (NLS‐GFP) after the sonoporation process, accounting for nuclear transfection. However, the low transfection efficiency, complex setup, and nontrivial glass capillary preparation should be addressed to be adopted by the field.

#### 3.5.2 Laser‐Assisted Cavitation

As an alternative, a new method was proposed that exploits the cavitation effect through laser pulses for the generation and control of microbubbles in a single step. Le Gac et al. reported the sonoporation of suspended cells with laser‐induced single bubble cavitation in a microfluidic chip.^[^ ^118^ ^]^ Microbubbles were generated by a frequency‐doubled pulsed laser (Nd:YAG laser). The PDMS microfluidic chip was fabricated using standard photolithography and dry etched silicon molds, and a human leukemia (HL60) cell suspension containing Trypan blue or calcein was injected into a microchamber. When a laser pulse was irradiated, a microbubble was nucleated in the chamber, and then the bubble‐induced flow (microjetting) exerted a shear force on the cells, leading to the rupture of the cell membrane and allowing uptake of dispersed dyes. Although this study was the first sonoporation using a single cavitation bubble on a microscale for intracellular delivery, limited throughput and low cell controllability were considered drawbacks. In a follow‐up study, the same group employed a microstructure array to trap cells, aiming at a higher level of controllability to enable precise intracellular delivery (Figure 9e).^[^ ^119^ ^]^ After the cells were injected and trapped in a PDMS microchip, microbubbles were generated in the vicinity of the trapped cells using a pulsed laser.^[^ ^118^ ^]^ In this study, high‐speed microscopy was used to visualize the process of microbubble generation and destruction which caused cell membrane perforation and transportation of Trypan blue into myeloma cells. As such, the study accomplished high precision of cavitation for sonoporation to investigate the membrane perforation mechanism of a single cell using high‐speed imaging. However, the throughput of the system was still low, and only small molecules could be internalized.

To address these issues, Wu et al. recently reported a large cargo delivery platform based on laser‐assisted microbubble cavitation.^[^ ^120^ ^]^ The cells were adhered to a porous SiO_2_ transmembrane with holes coated with titanium in a crescent‐shape on the side wall (Figure 9f). The SiO_2_ membrane was placed above an array of vertical silicon channels, providing fluid passages for macromolecule delivery. Upon laser illumination, titanium‐induced heating and vaporization triggered inertial cavitation of microbubbles for lipid membrane disruption.^[^ ^121^ ^]^ Concurrently, the fluid in the elastic chamber was pressurized to actively transport cargos into the permeabilized cells. Harnessing this unique approach, the delivery efficiency of 40 kDa dextran reached 90% for primary normal human dermal fibroblasts (NHDFs) and human primary renal proximal tubule epithelial cells (RPTECs) and 60% for human peripheral blood monocyte‐derived macrophages (PB‐MDMs). Moreover, living bacteria and antibiotics were successfully introduced into NHDF cells while maintaining their functionality. This study demonstrates the possibility of using sonoporation for transporting various macromolecules with a decent throughput (≈100 000 cells min^−1^); however, the system required highly laborious device fabrication and integration as well as complicated operation procedures. Moreover, suspension cell line could not be processed.

## 4 Electroporation for Intracellular Delivery

It is an indisputable fact that electroporation is one of the leading intracellular delivery methods. Electrical membrane perforation was first reported in 1958,^[^ ^122^ ^]^ and the technique has been successfully commercialized; however, there are several fundamental drawbacks as well. Before discussing how microfluidics has been synergistically integrated with electroporation, we will first briefly introduce the basic working mechanism considering its significance and associated concerns.

### 4.1 Mechanisms of Electroporation

Electroporation is a technique in which an electrical field is applied across a cell to permeabilize the cell membrane, allowing foreign cargos to be delivered into cells.^[^ ^123^ ^]^ Briefly, membrane discontinuities are generated by electroporation in two steps when the applied potential difference across the membrane exceeds the critical voltage.^[^ ^124^ ^]^ First, the thermal fluctuation of lipid molecules leads to the formation of sub‐nanopores, called hydrophobic pores, in the cell membrane.^[^ ^125^ ^]^ Next, the continued electric field expands the hydrophobic pores (>2 nm) by perturbing the water molecules and tilting the hydrophilic lipid heads. Consequently, enlarged nanopores called hydrophilic pores are created, allowing the introduction of external molecules into the cytoplasm via electrophoresis.^[^ ^125^ ^]^ Technically, the nanopore formation phenomena by electroporation can be optimized by modulating the pulse duration, frequency, and voltage to achieve a high level of intracellular delivery of external cargos. As electroporation exhibits relatively consistent delivery performance across cell types, it has become one of the most popular intracellular delivery strategies.

### 4.2 Bulk Electroporation

#### 4.2.1 Cuvette Electroporation

**Figure 10:**
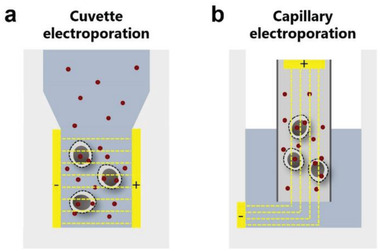
Schematics of electroporation: a) in a cuvette (also called bulk electroporation) and b) a capillary (also called capillary electroporation). Red dots indicate the target material.

Electroporation gained prominence as an attractive gene‐editing method after Neumann et al. transfected mouse L cells with plasmid and linear DNA in 1982.^[^ ^126^ ^]^ After this pioneering work, electroporation was employed for the transfection of eukaryotic and prokaryotic cells, including lymphocytes,^[^ ^127^ ^]^ plant cells,^[^ ^128^ ^]^ embryonic stem cells,^[^ ^129^ ^]^ *Escherichia coli*,^[^ ^130^ ^]^ and *Lactococcus lactis*.^[^ ^131^ ^]^ Most of the early procedures were performed using a cuvette; thus, the cell suspension was processed in bulk.^[^ ^128^, ^130^, ^131^ ^]^ As illustrated in **Figure** **10**a, the conductive buffer containing cells and cargos was placed in a cuvette, and two parallel electrodes were placed to generate an electrical field. Although the early electroporator showed its applicability to diverse cell types, the electrical condition of the continuous high‐voltage electric field was suboptimal and could not yield maximum delivery. Thus, a high‐voltage electric field and high‐voltage pulses with short durations (10–20 µs) were first used to induce efficient membrane permeabilization.^[^ ^132^ ^]^ Then, a lower voltage with a longer pulse (≈10 ms) was applied again for the transport of molecules into the cytoplasm via electrophoresis.^[^ ^132^, ^133^ ^]^ A higher transfection rate was later reported by optimizing the electrical parameters such as pulse intensity, duration, number, and time interval between pulses.^[^ ^132^, ^133^, ^134^, ^135^ ^]^

#### 4.2.2 Fundamental Challenges of Bulk Electroporation

Although bulk electroporation has become a daily use intracellular delivery technique in the laboratory, several critical problems should be noted. In general, bulk electroporation exploits extremely high voltage to reach the critical threshold of the electric field for cellular membrane perturbation.^[^ ^124^, ^128^ ^]^ When such a high operation condition is applied to a large cuvette system compared to cell size, a nonuniform electric field is presented, causing inconsistent and excessive cell perturbation and imposing high cytotoxicity. Additionally, it is well known that the applied voltage causes electrolysis, ohmic heating, metal ion contamination, and pH change, which alter the characteristics of buffer solutions and adversely affect the stability of both cells and biomolecules.^[^ ^136^, ^137^, ^138^, ^139^, ^140^ ^]^ In particular, electroporation of primary cell lines, which is important for therapeutic applications, critically suffers from electroporation‐induced cell toxicity,^[^ ^141^, ^142^ ^]^ low long‐term viability, and delayed recovery.^[^ ^57^, ^143^ ^]^ Thus, there have been a number of attempts to deal with these hurdles, and among them, a capillary, a form of a microchannel, has been proposed for safer and more effective electroporation.^[^ ^144^, ^145^ ^]^

#### 4.2.3 Capillary Electroporation

To overcome the issues associated with cuvette‐based bulk electroporation described above, capillary electroporation was developed with a smaller layout.^[^ ^144^ ^]^ As can be seen in Figure 10b, Kim et al. introduced a capillary with a diameter of 0.65 mm and a length of 30 mm for electroporation. The capillary was filled with a cell suspension mixed with external cargos, and an electrode (anode) was manually inserted into the capillary.^[^ ^144^ ^]^ Unlike bulk cuvette electroporation, the cathode is positioned outside the capillary separating the cathode and anode, but the electrodes are connected through electrolytes. The anode was connected to a pulse generator, and high‐voltage (0–2500 V) square wave pulses were applied for electric cell perturbation. By locating the cathode outside the capillary, the adverse effects resulting from electrical cell perturbations could be reduced, yielding higher cell viability.^[^ ^146^ ^]^ Since the presented layout also provided a localized, reduced, and uniform electric field in the capillary, higher transfection efficiency for hard‐to‐transfect cells, such as human mesenchymal stem cells (hMSCs), was demonstrated.^[^ ^144^ ^]^ Furthermore, the capillary geometry allows low‐volume electroporation operation (10 µL), which could be an economical solution for situations dealing with a small volume of cell samples or costly cargos. However, the platform is extremely limited in throughput (less than millions of cells per run), restricting its possibility for adoption in cell therapy applications. Additionally, the requirement of costly pipettes containing electrodes and special buffer solutions significantly lowers its practicability. Note that delivery efficiency and viability of electroporation strongly depend on buffer composition and cell type.^[^ ^147^ ^]^

### 4.3 Microfluidic Electroporation

The capillary geometry has naturally evolved to explore other microfluidic layouts. By going small, highly localized, concentrated, and uniform electrical fields can be generated; thus, electroporation can be inherently performed at lower voltages, where increased cell viability and uniform delivery can be expected. Microfluidics also offers higher flow controllability for precise and gentle cell manipulation, which potentially allows robust intracellular delivery with a scalability not fully available from capillary electroporation. In addition, via microfluidic integration, single‐cell level real‐time monitoring is available for underpinning the electrical membrane disruption phenomenon. Accordingly, a number of microfluidic electroporation platforms have been reported.^[^ ^148^ ^]^ The microfluidic electroporation approaches can be classified into 1) static (Section 4.3.1) and 2) flow‐through electroporation (Section 4.3.2), depending on cell motion during electroporation.

#### 4.3.1 Microfluidic (and Nanofluidic) Static Electroporation

##### Microfluidic Cell Trapping‐Based Electroporation

To improve electroporation performance and cell integrity, a pioneering study of single‐cell microfluidic electroporation was reported by Huang and Rubinsky in 1999.^[^ ^149^ ^]^ In this study, a silicon nitride membrane with microscale holes was vertically sandwiched between two silicon layers containing electrodes. The cell suspension was injected into the upper channel by a syringe pump, and then the cells were trapped in the microholes on the silicon nitride membrane. Because the two electrodes were within a few micrometers of each other, a low direct current (DC) voltage (≈10 V) was applied for membrane permeabilization of the trapped cells. Although this study focused on biophysical investigations by sweeping different electric parameters and conditions, the same group later used the platform mainly for intracellular delivery.^[^ ^150^ ^]^ Through this microfluidic electroporation approach, successful delivery of YOYO‐1 dye and EGFP encoding plasmid DNA into prostate adenocarcinoma ND‐1 cells was demonstrated; however, laborious fabrication and complex integration are the main weaknesses of the system.

**Figure 11:**
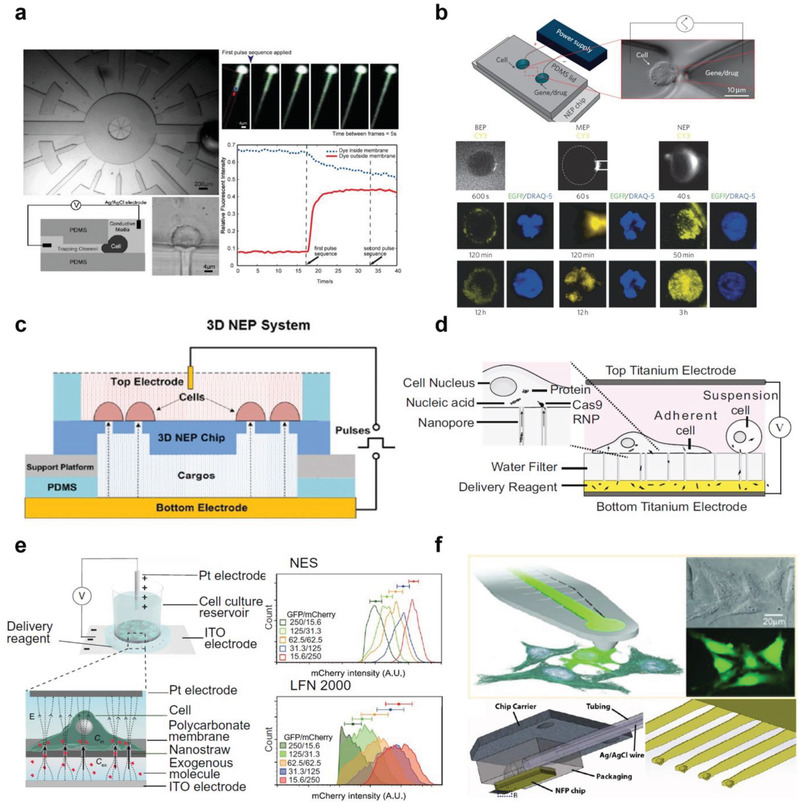
Microfluidic static electroporation. a) Single cell‐based electroporation setups. Reproduced with permission.^[^ ^151^ ^]^ Copyright 2005, Royal Society of Chemistry. b) Nanochannel electroporation for rapid biomolecule delivery. Reproduced with permission.^[^ ^163^ ^]^ Copyright 2011, Springer Nature. c) Electroporation using a silicon nanohole array for adherent cells. Reproduced with permission.^[^ ^167^ ^]^ Copyright 2016, Royal Society of Chemistry. d) Nanopore electroporation with a track‐etched polycarbonate membrane for adherent and suspension cells. Reproduced with permission.^[^ ^170^ ^]^ Copyright 2019, National Academy of Sciences. e) Electroporation using nanostraws protruding from the track‐etched polycarbonate membrane. Reproduced with permission.^[^ ^176^ ^]^ Copyright 2018, American Association for the Advancement of Science. f) Nanofountain probe tip enabled electroporation using an atomic force microscope setup. Reproduced with permission.^[^ ^181^ ^]^ Copyright 2013, American Chemical Society.

In another study, Khine et al. reported a single‐cell electroporator with a planar design on a PDMS–glass chip.^[^ ^151^ ^]^ The platform adopted a hydrodynamic cell trap design in which the cells were immobilized by the negative pressure applied to the trapping channel (**Figure** **11**a). Ag/AgCl electrodes were placed, and a very low electric potential (<1 V) was applied for the electropermeabilization of cells. Owing to the resistance difference between the main channel and low trapping channel, the highest potential drop was created in the cell trapping zone, effectively generating cell membrane discontinuities. The internalization of Trypan blue and calcein AM in HeLa cells was demonstrated. In a follow‐up study, the same group reported a microfluidic electroporator interfaced with a 96‐well plate to provide an independent electroporation environment for trapped cells.^[^ ^152^ ^]^ The entire electroporation process was controlled and monitored in each well, enabling real‐time feedback control of electroporation conditions. As another strategy, electrophoresis was employed to further assist electroporation.^[^ ^153^, ^154^ ^]^ For instance, after cell trapping, an electric potential (300 mV) below the perforation threshold was applied to electrophoretically concentrate cargos in the vicinity of the cell.^[^ ^153^ ^]^ Cells were then permeabilized by electroporation and a low electric field was again applied to electrophoretically transport the concentrated cargos through the generated discontinuities on the membrane. Using this subsequent delivery sequence, expedited delivery of calcein and 70 kDa Oregon green into HeLa cells was demonstrated. However, no delivery of functional materials, such as plasmid DNA, was reported. Valero et al. later processed primary human MSCs and mouse myoblastic C2C12 cells in a similar fashion.^[^ ^155^ ^]^ The cells were transfected with EGFP‐ERK1 (extracellular signal‐regulated kinase) encoding plasmids, and cell functionality was assessed by intracellular protein dynamics. Approximately 70% of C2C12 cells and 90% of hMSCs were successfully transfected via microfluidic electroporation. Nevertheless, only significantly low cell numbers could be processed per run (less than 50 cells).

As another strategy, membrane sandwich electroporation (MSE) platform has been reported to improve electrotransfection performance.^[^ ^156^, ^157^, ^158^ ^]^ Fei et al. demonstrated microfluidic cell trapping electroporation using two track‐etched polyethylene terephthalate (PET) porous membranes.^[^ ^156^ ^]^ The cells were trapped on a nanoporous membrane by vacuum, and a microporous PET membrane was added on top of the trapped cells. The authors claimed that cell immobilization on a porous surface led to localized cell electroporation, allowing the use of a low applied voltage (35 V cm^−1^) to preserve cell integrity, and the additional top membrane improved gene transfection performance due to the facilitated DNA migration phenomenon. Consequently, more than 90% of NIH 3T3 cell viability was achieved using a much lower electric field (35 V cm^−1^) compared with a single cuvette‐based system (1600 V cm^−1^). In addition, MSE exhibited higher transfection efficiency of two plasmid DNAs encoding GFP (5.7 kbp) and secreted alkaline phosphatase (SEAP) (6.6 kbp) than the conventional cuvette style electroporation, as assessed using fluorescence imaging and protein activity assays, respectively. Following that, the same group presented a similar strategy by employing converging nozzle channels in which mouse ESCs were transfected using pmaxGFP (3.5 kbp) and gWiz SEAP (6.6 kbp) plasmid vectors.^[^ ^157^ ^]^ Note that several other studies have also investigated micropores^[^ ^159^, ^160^ ^]^ and microwells coated with microelectrode arrays^[^ ^161^, ^162^ ^]^ within the microchip for electrotransfection of seeding cells.

Regarding the intracellular delivery performance of microfluidic electroporation, the studies discussed here exhibited several advantages, including the use of significantly lower voltage and the introduction of a highly concentrated and uniform electric field, which enabled less invasive and more effective transfection/delivery compared with bulk electroporation. However, one of the main limitations of this technique is its low scalability, which is not ideal for processing large volumes of samples, thus limiting its impact toward cell‐based therapy.

##### Nanochannel Electroporation

Nanochannel electroporation has several advantages over conventional bulk and microchannel‐based electroporation. For example, the nanochannel itself can serve as a delivery pathway for transporting external molecules into the cytoplasm, and the amount of cargo can be precisely modulated by tuning the electrical parameters. Furthermore, a highly localized electric field within a nanochannel can mitigate cytotoxicity, substantially improving cell viability compared with bulk/microelectroporation.

In 2011, the Lee group reported nanochannel electroporation capable of high reagent dosage control and rapid transport of foreign cargos (Figure 11b).^[^ ^163^ ^]^ To create a nanochannel 90 nm in diameter, the DNA combing and imprinting (DCI) method^[^ ^164^ ^]^ was employed. The optical tweezer system was also used to position a cell at the tip of the nanochannel, and electroporation was performed with voltages ranging from 150 to 350 V, without affecting cell viability. Consequently, rapid injection (<30 ms) of PI into K562 cells, which is significantly faster than bulk and microfluidic electroporation (≈150 s), was observed through fluorescence microscopy. The study claimed that the rapid delivery of nanochannel electroporation was possible because of the strong electrophoresis induced by the concentrated electric field at the nanojunction. To validate this, the authors demonstrated the transfection of 3.5 kbp GFP plasmid DNA, and GFP expression was observed within 6 h; faster than that of bulk (24 h) and microchannel (20 h) electroporation. Furthermore, to demonstrate dosage controllability, Cy3‐labeled 18‐mer oligodeoxynucleotide (ODN) was delivered into Jurkat cells as a function of pulse duration, and a monotonic increase in Cy3 fluorescence intensity was observed. Molecular beacons, siRNA, and quantum dots were introduced via nanochannel electroporation. This study demonstrated the benefits of using a nanochannel for electroporation, which enabled rapid and well‐controlled intracellular delivery.

A number of following studies have utilized nanopore array patterned substrates to deliver diverse reagents, including plasmid DNA, micro RNA, and molecular beacon via nanochannel electroporation.^[^ ^165^, ^166^, ^167^, ^168^, ^169^ ^]^ Among them, a notable 3D nanochannel electroporation platform was developed (Figure 11c),^[^ ^167^ ^]^ partially overcoming the previous low throughput issues in nanochannel electroporation design.^[^ ^163^ ^]^ A silicon wafer was etched to construct an array of vertical flow‐through nanochannels with diameters of hundreds of nanometers. The silicon structure was then bonded with a support, a PDMS spacer, and a bottom electrode. Approximately 40 000 cells were seeded above the nanochannel array, and the cargo was electrokinetically transported through the bottom channel by applying an electrical field. Using the platform, PI dye was uniformly delivered into H9C2 cells at precisely controlled doses. As a key application, large weight OSKM plasmid DNA (≈13 kbp) labeled with green fluorescence was delivered into mouse embryonic fibroblast (MEF) cells, and approximately twofold higher transfection efficiency (with 90% of cell viability) was exhibited with nanochannel electroporation compared with bulk electroporation. In this study, nanochannel electroporation was scaled up from tens of cells per run to tens of thousands of cells per run.

Cao et al. recently presented nanopore electroporation using a track‐etched polycarbonate (PC) membrane containing nanopores with 100 nm diameters.^[^ ^170^ ^]^ Although the approach was very similar to that previously reported,^[^ ^167^ ^]^ the main distinction was the utilization of surface coating proteins, such as fibronectin and poly‐l‐lysine, on the PC membrane to facilitate tight adhesion/contact of cellular membrane to the surface regardless of adherent or suspension cell type (Figure 11d). Approximately 5000–15 000 cells were seeded and cultured overnight on the membrane before electroporation. Titanium electrodes on the bottom and top plates were used to transport external molecules from the bottom chamber into cells through nanopores. Several cell types, including HeLa, HEK293, 3T3, and Jurkat cells, were evaluated for mCherry mRNA and GFP plasmid transfection. Maximum nucleic acid transfection efficiency of 80% and cell viability greater than 95% were exhibited using HeLa cells.

As another nanochannel electroporation strategy, Yun et al. recently developed a nanoinjection system.^[^ ^171^ ^]^ The system comprises several micro‐ and nanochannels for cell loading, harvesting, and injection. After the cells were pumped into the loading chamber, each cell was individually positioned in the injection region via fluidic control. The trapping region was connected to a nanochannel where an electric field was first applied to permeabilize the membrane and then the external molecules were injected into the trapped cell via electroosmosis. One of the main advantages of this approach is the high controllability of synchronization between cell positioning and cargo injection, with high dosage control. Using the platform, 26 kDa red fluorescent protein (RFP) and GFP plasmid DNA (5 kbp) were delivered into hMSCs, exhibiting 24–51% DNA transfection efficiency with viability greater than 95%. Although the system demonstrated improved controllability, scalability is still low, and a highly complex setup is another major concern. Furthermore, the system requires costly fabrication using a femtosecond laser and equipment to operate, and the channel clogging issue needs to be addressed for robust performance.

Nanochannel electroporation evidently has a strength in high delivery efficiency, minimal cellular damage, and precise dosage control (i.e., consistent delivery), with the possibility of scale‐up to high throughput processing.^[^ ^169^ ^]^ However, the platform is intrinsically limited in scalability owing to a noncontinuous process, even with channel parallelization. In addition, labor‐intensive chip fabrication when creating nanochannels and the additional apparatus required for electrical controls add complexity, making the system less attractive. Difficulties in delivering large nanoparticles due to dimensional constraints and non‐negligible Stokes drag (i.e., poor particle migration) is another challenge that should be addressed.^[^ ^172^ ^]^

##### Nanostraw Electroporation

Nanochannel electroporation with a free‐standing hollow nanostraw array was substantially investigated by the Melosh group.^[^ ^173^, ^174^, ^175^, ^176^ ^]^ As shown in Figure 11e, an array of nanochannels (e.g., nanowires), termed nanostraws, with a diameter of ≈250 nm and a height of 1.5 µm, constructed by RIE of a track‐etched PC membrane, was utilized for electroporation.^[^ ^174^, ^176^ ^]^ The nanostraw array was then bonded with a PDMS chip, and the cells were seeded prior to electroporation. Below the membrane, there was a microfluidic channel for the injection of target biomolecules through the nanostraws. An indium tin oxide (ITO) electrode plate at the bottom and a Pt electrode were placed in the cell culture chamber, and a voltage (<60 V) was applied for cell membrane permeabilization. Harnessing the unique layout, RFP encoding plasmid DNA (4.7 kbp) was successfully transfected into CHO cells (≈80%) and HEK293 cells (67%) with high viability of both cells (>98%). Furthermore, cotransfection of two plasmid DNAs (encoding RFP and GFP) was also demonstrated with 74% efficiency. After this initial report, the system was developed further for cytosol extraction^[^ ^177^ ^]^ and primary cell applicability with consistent delivery.^[^ ^176^ ^]^ In the latter study, HEK293 cells were transfected with mCherry mRNA using nanostraw electroporation, and the results were compared with those of lipofection (Lipofectamine 2000). The transfection efficiency of HEK293 cells using nanostraw electroporation (75–90%) was higher than that obtained using lipofection (50–70%); greater uniformity in delivery (i.e., consistent delivery) was also observed. Additionally, diverse primary cell types, including human iPSC‐derived cardiomyocytes, human embryonic stem cells, fibroblasts, and mouse primary glial cells, were transfected with EGFP mRNA, achieving 60–80% delivery efficiency. However, one of the major concerns of using nanostraw electroporation is the potential adverse effects of culturing cells on nanowires^[^ ^71^, ^72^ ^]^ on their long‐term viability and functionality, especially for sensitive primary cells.

Recently, Schmiderer et al. reported a modified nanostraw electroporation system for transfecting suspension cells.^[^ ^142^ ^]^ A centrifuge was used to adhere HSPCs to the nanostraw array. Diverse molecules, including PI, GFP mRNA (≈77%), siRNA, oligonucleotides (≈80%), and dextran (61–83%), were delivered into the cells. Furthermore, the functionality of transfected HSPCs was validated both in vitro and in vivo using gene expression analyses and mouse engraftment, respectively. Other vertical nanostructures (e.g., nanotubes and nanoflowers) have also been utilized for electroporation in a series of studies.^[^ ^178^, ^179^, ^180^ ^]^ Nonetheless, the limited throughput and laborious nanodevice fabrication remain fundamental drawbacks.

##### Nanofountain Electroporation

As another format, static electroporation, was performed using nanoprobes integrated with a conventional atomic force microscope (AFM).^[^ ^181^, ^182^, ^183^, ^184^ ^]^ The key feature of the system, termed nanofountain, is the integration of microchannels embedded in a cantilever (Figure 11f).^[^ ^181^ ^]^ The solution containing external cargos can be injected into cells through the hollow nanoprobe tips. After the probe tip was positioned in contact with the target cell by the AFM controller, an electric field was applied to permeabilize the membrane; therefore, external biomolecules were injected into the cytoplasm through the embedded channel via an external microfluidic pump. The small diameter of tips, ranging from 1 to 100 nm, enabled the delivery of accurate amounts of cargo into cells and minimized damage to the cells during permeabilization.^[^ ^185^ ^]^ In a follow‐up study harnessing the improved precision of electroporation by nanofountain technology, single HT1080 cell transfection with two different biomolecules was reported over time.^[^ ^182^ ^]^ CRISPR‐Cas9 transfection using HEK293 cells was also conducted to generate a monoclonal cell line.^[^ ^183^ ^]^ Note that intracellular delivery can also be achieved by injecting cargos through the pores created by the AFM probe tip without applying an electrical field (i.e., no electroporation‐based cell membrane permeabilization), similar to the microinjection strategy.^[^ ^186^, ^187^ ^]^

As discussed above, the major benefit of nanofountain systems is the ability to precisely control the probe tip on the target cell(s) using the existing AFM setup, and to monitor the electroporation process. Furthermore, the low voltage and concentrated electric field assured high cell viability and uniform delivery compared with bulk electroporation. However, the system is not able to process suspension cell lines, suffers from extremely low scalability, and requires costly AFM and burdensome fabrication, which lowers its wider usage.

#### 4.3.2 Microchannel Flow‐Through Electroporation

Electroporation can also be performed when cells are in motion instead of stationary. The major benefit of flow‐through electroporation is the ability to process a large number of cells in a continuous manner, opening the possibility of its application in cell‐based therapies. In the following sections, we discuss the development of high throughput microfluidic intracellular delivery via flow‐through electroporation.

##### Constant Flow‐Through Electroporation

Lin et al. introduced a pioneering study on a flow‐through electroporation microchip in 2001.^[^ ^188^ ^]^ Two gold parallel plate electrodes were placed on the top and bottom of the PMMA microchannel, and cells passing through the electrode region were permeabilized with an electric field. Human hepatocellular carcinoma cells mixed with *β*‐galactosidase and GFP plasmid (pCMV‐LacZ; 7.2 kbp) were processed under different conditions of varying flow rates, pulse durations, and intensities. Although the transfection efficiency was not directly reported, this study showed that continuous electroporation was possible within the microfluidic system, suggesting that high throughput electroporation can be realized.

**Figure 12:**
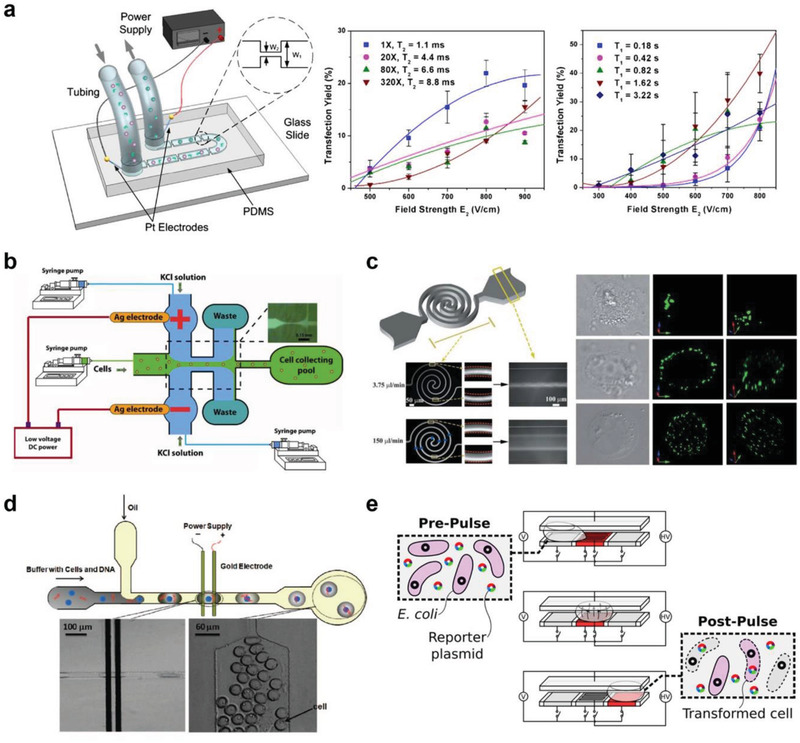
Microfluidic flow‐through electroporation. a) Schematic of the microfluidic electroporation device with a constriction and optimization. Reproduced with permission.^[^ ^191^ ^]^ Copyright 2010, Elsevier. b) Flow‐through electroporation assisted by flow focusing for high electric field gradient. Reproduced with permission.^[^ ^202^ ^]^ Copyright 2010, Springer Nature. c) Uniform flow electropermeabilization with Dean flow. Reproduced with permission.^[^ ^204^ ^]^ Copyright 2010, Royal Society of Chemistry. d) Droplet‐based flow‐through electroporation. Reproduced with permission.^[^ ^213^ ^]^ Copyright 2009, American Chemical Society. e) Schematic of droplet electroporation in the serpentine channel and improved transfection efficiency of microalgae. Reproduced with permission.^[^ ^216^ ^]^ Copyright 2017, American Chemical Society.

After this initial study, the Lu group reported a constriction microchannel design for flow‐through electroporation by applying DC and alternating current (AC) voltages.^[^ ^189^, ^190^, ^191^, ^192^, ^193^, ^194^, ^195^, ^196^ ^]^ The key feature of the design involves concentrating the DC electric field (400 V cm^−1^) in the bottleneck region, which allows effective and uniform cell electroporation.^[^ ^189^ ^]^ Impermeable SYTOX green dye was used to characterize cell permeabilization, and 58% of CHO cells showed fluorescence signals, with cell viability higher than 80%. Later, the same group achieved a 21.2% pEGFP‐C1 plasmid DNA transfection yield for CHO cells with a modified channel layout.^[^ ^190^ ^]^ A new protocol was established to improve transfection efficiency by thoroughly optimizing several operating conditions (**Figure** **12**a).^[^ ^191^ ^]^ Using this approach, a maximum of 75% plasmid DNA (pEGFP‐C1) transfection efficiency of CHO cells was achieved. Note that a high throughput intracellular delivery processing rate (10^7^ cells min^−1^) was demonstrated through constant flow‐through electroporation.^[^ ^191^ ^]^

As another approach, Kim et al. introduced hydrogel plugs to create a focused electric field in a PDMS–glass microchannel.^[^ ^197^ ^]^ Conductive hydrogel plugs, termed “salt bridges,” were constructed to serve as an electric pathway, generating 0.9 kV cm^−1^ across the main channel with a DC voltage of 10 V. Cells were injected into the channel with cargos, and flow‐through electroporation was performed when the cells passed the salt bridge region. Following this method, 60% of K562 cells were permeabilized with 80% viability. Transfection of pEGFP‐C2 plasmid DNA (4.7 kbp) into K562 cells was also demonstrated at a processing rate of 10^5^ cells min^−1^, although no quantitative transfection efficiency was reported.

Instead of direct current, AC has also been utilized for flow‐based electroporation to detour electrolysis issues (e.g., bubble evolution^[^ ^198^ ^]^). Ziv et al. used a pulse generator to apply AC through tungsten wires connected to the inlet and outlet of the device.^[^ ^199^ ^]^ The murine MSCs were transfected with pGFP‐N1 plasmid DNA (4.7 kbp), and the delivery was characterized by fluorescence microscopy (no quantitative data was reported).^[^ ^199^ ^]^ To improve delivery performance, the AC electrical field conditions were modulated. Adamo et al. designed a comb pattern of electrodes, enabling repeated flow‐through electroporation.^[^ ^200^ ^]^ Using a PDMS–glass chip, 10–30 sets of gold electrodes were patterned on the channel bottom to generate uniform AC fields. Through the system, 80% of 10 kDa fluorescein‐conjugated dextran was delivered into HeLa cells with 85% viability. Furthermore, GFP silencing siRNA was transfected into HeLa cells, albeit at a lower knockdown efficiency (20–50%) compared with lipofection (60%). As another method, dielectrophoretic (DEP) sorting was also integrated with flow‐electroporation to separate viable and nonviable cells.^[^ ^201^ ^]^ Diverse cell types, including primary human umbilical vein endothelial cells (HUVECs) and primary human skin fibroblast (HSF) cells were transfected with 4.7 kbp EGFP plasmid DNA, albeit at low efficiency (≈30%).

While AC‐based flow‐through electroporation exhibited strength in mild polarization and could be free from electrolysis, a tedious optimization process for identifying the optimal operation condition and additional apparatus (i.e., pulse generator) should be accompanied for each cell type. Furthermore, detailed studies investigating cell functionalities and stabilities should be explored for cells treated with flow electroporation.

##### Flow‐Through Electroporation Assisted by Hydrodynamic Effects

As discussed above, electrolysis has become a significant issue for DC‐based microfluidic flow‐through electroporation. As a solution, hydrodynamic focusing was employed to minimize bubble formation, heat shock, pH change, and hydrolysis. Zhu et al. employed sheath fluids to separate the cell suspension stream from the electrodes (Figure 12b).^[^ ^202^ ^]^ KCl conductive buffer solution and cell suspension were injected through the sheath and core inlets, respectively. The core stream of the cell suspension was hydrodynamically focused by the sheath fluids, and the width of the core fluid stream was modulated by the relative flow rates. By having a thin core fluid (≈5 µm), an electronic field higher than 1.25 kV cm^−1^ was achieved only by applying 1.5 V. Under this condition, 70% of yeast cells were permeabilized in a high throughput manner (10^4^ cells min^−1^). Although the system only processed cells with a diameter of 3–4 µm for nonfunctional cargo delivery, the study demonstrated potential for high throughput flow‐focusing electroporation. The approach was later improved using a multisheath to enable high functional biomolecule internalization. Using multistream, 70–90% of plasmid DNA (pEGFP‐C3, 4.7 kbp) transfection efficiency was demonstrated using HEK293, Neuro‐2A, C2C12, and PC12 cells.^[^ ^203^ ^]^ However, despite efforts to detach electrodes from cells, the low cell viability (55–75%) should be addressed for further applications.

Another key hydrodynamic phenomenon utilized for microfluidic flow‐through electroporation is the flow vortex. A spiral microfluidic channel was used to employ Dean flow effects during electroporation, as shown in Figure 12c.^[^ ^204^ ^]^ It was claimed that the uniform electric field in a straight microchannel electroporation system can only create membrane pores in both poles of the membrane because there was no transverse motion of cells. Thus, by having Dean flows that can rotate the cell rigorously, theoretically, the entire cell membrane was expected to be perforated via electroporation, potentially achieving higher levels of intracellular delivery. The hypothesis was validated by imaging CHO cells transfected with YOYO‐1‐labeled plasmid DNA (pEGFP‐C1; 4.7 kbp), where a uniform distribution of fluorescence signals was observed. Furthermore, greater plasmid DNA transfection efficiency (≈30%) and slightly higher viability (≈80%) were demonstrated in the spiral channel than in the straight channel; however, low transfection efficiency was still observed. Recently, a series of studies reported flow‐through electroporation with sheath flow for the transfection of primary human T cells with and without acoustic manipulation.^[^ ^205^, ^206^ ^]^ In the latter case, the throughput of the system was improved to 10^7^ cells min^−1^ while successfully transfecting T cells with mCherry mRNA (75–95%).^[^ ^206^ ^]^

In other studies, vortex‐assisted electroporation^[^ ^53^, ^207^, ^208^ ^]^ was proposed to address the difficulty of precise multigene and/or multimolecular delivery using conventional bulk and capillary electroporation. This approach utilizes the inertial cell migration phenomenon^[^ ^50^ ^]^ in vortices developed in expansion–contraction trapping chambers.^[^ ^209^ ^]^ Electroporation was performed once the cells were trapped in the chambers.^[^ ^210^ ^]^ Solutions with different cargos were sequentially injected for multicargo delivery, while the cells were hydrodynamically trapped in the chamber during solution exchange. Using MDA‐MB‐231 cells, PI dye and calcein AM were internalized to assess delivery efficiency (70–92%) and viability (58–94%), respectively.^[^ ^210^ ^]^ The authors also demonstrated that two different plasmid DNAs (pQCXIP‐NLS‐Vx3‐mEGFP; 7.9 kbp and pcDNA3‐mRFP; 6.1 kbp) could be delivered into MDA‐MB‐231 cells. The system enables sequential multicargo delivery and on‐chip washing; however, the approach was limited in throughput (20 cells per run) and very low transfection efficiency and required excessive amounts of cargo materials for delivery.

##### Droplet Electroporation

Droplet‐based microfluidics have shown great promise in various biomedical applications,^[^ ^211^ ^]^ and their use has been expanded to electroporation as well. The first demonstration used droplets containing fluorescein dye and yeast cells, where the dye was internalized into cells by applying an electrical field to the droplets.^[^ ^212^ ^]^ Although the study first reported the idea of droplet electroporation, it mainly characterized the size and ion concentration change of droplets during the process of electroporation, rather than investigating delivery performance. Later, Zhan et al. demonstrated the delivery of plasmid DNA (pEGFP‐C1; 4.7 kbp) into CHO cells via microfluidic droplet electroporation.^[^ ^213^ ^]^ Using a PDMS T‐junction microchannel, a continuous phase of hexadecane with a disperse phase of electroporation buffer with cells and cargos was used to generate microdroplets (Figure 12d). A constant voltage was applied to two gold electrodes patterned on the bottom substrate downstream for electroporation. Owing to the monodispersed droplet size, a uniform electric field was applied across the cells in the droplets. Furthermore, because of the extremely short distance between electrodes (≈20 µm), a low DC voltage (5–9 V) was required for electroporation, minimizing the risk of electrolysis. While a maximum transfection efficiency of 11% and cell viability of 68% were obtained with high scalability, the low efficiency compared with the nondroplet microfluidic electroporation was a drawback.

As another layout, Qu et al. utilized a serpentine microchannel for a chaotic mix of droplets containing microalgae cells and DNA to increase transfection efficiency during electroporation.^[^ ^214^ ^]^ After droplets were generated upstream, they migrated to the curved channel region where five pairs of parallel gold electrodes were deposited for repetitive electroporation. The transfection efficiency of microalgae with DNA fragments was two to three orders of magnitude higher than that of conventional bulk electroporation. It was claimed that the chaotic mixing of droplets facilitated DNA access to the cells, resulting in effective transfection performance. This mixing enhancement was further confirmed in a recent work by Li et al., who transfected K562 cells using cationic lipids within droplets flowing in a serpentine microchannel (without electroporation).^[^ ^215^ ^]^

A completely different droplet‐based electroporation strategy using electrowetting‐on‐dielectric (EWOD) digital microfluidics was also demonstrated (Figure 12e).^[^ ^216^ ^]^ A conventional EWOD layout was used, and for microbial transfection, a single droplet containing EcNR2 *E. coli* and plasmid DNA encoding antibiotic resistance genes was generated within the EWOD device. Via the EWOD control, the droplet was positioned over the electrodes for electroporation, and pulses were applied to the droplet. After the electropermeabilization of bacteria, the droplet was mixed with a recovery droplet to stabilize the electroporated cells. The transformation efficiency was defined by calculating the proportions of colonies that survived after antibiotic medium culture, and a higher transformation efficiency of 8.6 × 10^8^ cfu µg^−1^ was achieved compared with the traditional cuvette electroporation (≈10^7^ cfu µg^−1^). This study exhibited significant improvement in microbial transformation efficiency as droplet electroporation was performed on the EWOD platform. However, the droplet electrolysis issue, low scalability, and complex fabrication and operational schemes need to be addressed.

## 5 Outlook and Perspective

The primary goal of developing a novel intracellular delivery approach is to achieve delivery performance at levels higher than those of the current techniques, such as viral transduction, lipofection, and electroporation. Microfluidics‐ and nanofluidics‐based intracellular delivery strategies have made unprecedented leaps toward higher and consistent delivery efficiency and viability, lower costs, process standardization, elimination of complexities in processing via automation, and precise dosage control, and now their application is moving forward to clinical trials along with commercialization. For example, a clinical trial (conducted by SQZ Biotech, a spin‐out company) seeking the possibility of using cell squeezing technology for cancer immunotherapy is in Phase 1 (NCT04084951). To make a real impact by establishing a true next‐generation microfluidic intracellular delivery platform, the following considerations should be taken into account from the very beginning of platform development.

First and foremost, researchers should consider the practicability of the platform. As reviewed, a large variety of novel microfluidic solutions have been proposed; however, it is often extremely difficult for researchers outside of the microfluidics community to immediately adopt the method due to the complexity of the system. High levels of transfection performance can be expected from microfluidic integration, surpassing existing technologies in performance; however, the system frequently becomes too complex for physicians or biologists to use. It should be assumed that potential users will face difficulties in operating any microfluidic system without a proper automation process or training; hence, they are likely to stick with their current methods despite low delivery efficiency. Therefore, the platform must be either fully automated or extremely simple to use. For example, we recently reported a clogging‐free, single‐step microfluidic intracellular delivery platform operated with a single syringe pump without the need for costly external instruments such as a microscope, a voltage source, or a camera.^[^ ^49^ ^]^ Additionally, the PDMS–glass chip configuration offers a low‐cost solution, demonstrating feasibility of the platform.

**Figure 13:**
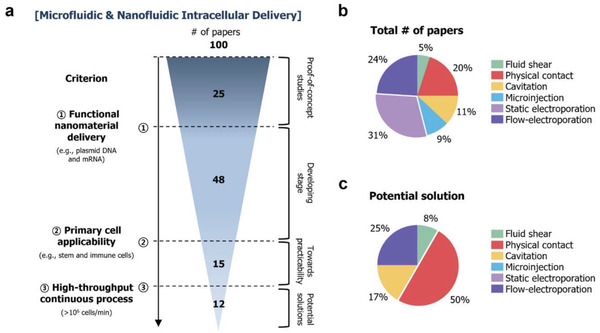
Analysis of publication on micro‐ and nanofluidic intracellular delivery in this review. a) Publication classification after applying three criteria, leaving only 12 microfluidic solutions. b) Publication classification based on delivery approach. c) Detailed analysis of the 12 remaining microfluidic approaches with high potential toward next‐generation intracellular delivery method.

The second consideration is the applicability of the system. We define applicability using the following three criteria: 1) the availability of functional nanomaterial delivery (ability to deliver materials that can actually engineer or alter cellular functions rather than pure fluorescence probe delivery), 2) primary cell applicability, and 3) high throughput and continuous process (high scalability for cell therapy). In this review, we have discussed 100 micro(nano)fluidics‐based intracellular delivery studies in depth (see **Table** **3**). After refining them based on these three criteria, only 12 articles remained, as shown in **Figure** **13**, where mechanical cell membrane disruption method and flow electroporation showing high potential to be employed by the field in the near future. Without platform applicability, the democratization of the newly developed method into a clinic or laboratory is hard to be expected. It should be mentioned that in the development of new microfluidic intracellular delivery strategies, the focus is often largely placed on technical originality because of publication. However, microfluidic researchers in academia should move forward by considering the platform applicability criteria along with pursuing system novelty.

**Table 3:** Summary of key microfluidic intracellular delivery techniques covered in this review

				Cargo type			
Mechanism	Method	Delivery principle	Cell type (primary cell)	Fluorescence probe	Functional nanomaterials/macromolecule	Throughput (cell processing rate)	Comments
Mechanoporation	Fluid shear	Shear force	DU145,^[^ ^45^ ^]^ NIE‐115,^[^ ^46^ ^]^ rat primary neuron ^[^ ^46^ ^]^	Calcein,^[^ ^45^ ^]^ FITC–dextran,^[^ ^45^ ^]^ FITC–BSA^[^ ^45^ ^]^	GFP pDNA^[^ ^46^ ^]^	N/A	–
		Vortex shedding	Human T cell ^[^ ^48^ ^]^	N/A	GFP mDNA^[^ ^48^ ^]^	10^7^ cells min^−1[^ ^48^ ^]^	Under commercialization by Indee Labs^[^ ^48^ ^]^
		Spiral vortex	HEK293,^[^ ^49^ ^]^ ES2,^[^ ^49^ ^]^ 3T3,^[^ ^49^ ^]^ KU812,^[^ ^49^ ^]^ HeLa,^[^ ^49^ ^]^ HCT116,^[^ ^49^ ^]^ MCF7,^[^ ^49^ ^]^ HDFa,^[^ ^49^ ^]^ K562,^[^ ^35^, ^49^ ^]^ MDA231^[^ ^35^, ^49^ ^]^	FITC–dextran^[^ ^35^, ^49^ ^]^	DNA nanotube,^[^ ^49^ ^]^ DNA donut,^[^ ^49^ ^]^ GFP mRNA,^[^ ^35^ ^]^ GFP pDNA,^[^ ^35^, ^49^ ^]^ GNP,^[^ ^35^ ^]^ DOX‐MSNs^[^ ^35^ ^]^	10^6^ cells min^−1[^ ^35^, ^49^ ^]^	Clogging‐free operation;^[^ ^35^, ^49^ ^]^ under commercialization by MxT Biotech^[^ ^35^ ^]^
	Constriction	Cell squeezing	HeLa,^[^ ^53^, ^54^, ^56^ ^]^ murine ESC,^[^ ^53^, ^56^ ^]^ NuFFs,^[^ ^53^ ^]^ murine dendritic cell,^[^ ^53^ ^]^ murine macrophage,^[^ ^53^ ^]^ murine B cell,^[^ ^53^, ^55^ ^]^ murine T cell,^[^ ^53^ ^]^ human T cell,^[^ ^57^ ^]^ Jurkat,^[^ ^62^ ^]^ K562,^[^ ^62^ ^]^ HEK293t^[^ ^62^ ^]^	FL‐dextran,^[^ ^53^, ^55^, ^56^, ^57^, ^62^ ^]^ FL‐protein^[^ ^53^, ^62^ ^]^	siRNA,^[^ ^53^ ^]^ GFP pDNA,^[^ ^56^ ^]^ CNT,^[^ ^53^ ^]^ GNP,^[^ ^53^ ^]^ Qdot,^[^ ^54^ ^]^ antigen,^[^ ^55^ ^]^ Cas9‐RNP^[^ ^57^ ^]^	10^6^ cells min^−1[^ ^53^, ^54^, ^56^, ^57^ ^]^	Under commercialization by SQZ Biotech (in Phase I trial; NCT04084951)^[^ ^53^ ^]^
		Deformation array	HEK293t,^[^ ^58^ ^]^ MCF7,^[^ ^58^ ^]^ SU‐DHL‐1,^[^ ^58^ ^]^ ES AB2.2,^[^ ^58^ ^]^ PC‐3,^[^ ^58^ ^]^ SUM159,^[^ ^58^, ^59^ ^]^ HeLa,^[^ ^58^ ^]^ HL60,^[^ ^59^ ^]^ SK‐BR‐3,^[^ ^59^ ^]^ human T cell,^[^ ^59^ ^]^ MDA231,^[^ ^58^, ^60^ ^]^ NIH 3T3,^[^ ^61^ ^]^ human ASC ^[^ ^61^ ^]^	FITC–ssDNA,^[^ ^58^ ^]^ FL‐dextran,^[^ ^59^, ^60^, ^61^ ^]^ FITC–siRNA^[^ ^59^, ^60^ ^]^	siRNA,^[^ ^58^, ^60^ ^]^ GFP pDNA,^[^ ^58^, ^61^ ^]^ CRISPR pDNA,^[^ ^58^ ^]^ Cas9 RNP^[^ ^58^, ^59^ ^]^	10^5^ cells min^−1[^ ^61^ ^]^	–
		Volume exchange	PC3,^[^ ^33^ ^]^ PBMC,^[^ ^33^ ^]^ K562,^[^ ^33^, ^34^ ^]^ HL60,^[^ ^34^ ^]^ HEY,^[^ ^34^ ^]^ HEK293,^[^ ^34^ ^]^ OVCAR‐3^[^ ^34^ ^]^	FITC–dextran,^[^ ^33^, ^34^ ^]^ RNA probe^[^ ^33^ ^]^	GFP mRNA,^[^ ^33^ ^]^ GFP pDNA,^[^ ^33^ ^]^ nanobead^[^ ^33^ ^]^	10^6^ cells min^−1[^ ^34^ ^]^	Under commercialization by CellFE^[^ ^33^ ^]^
	Poking	Cell poking	MDA231,^[^ ^75^ ^]^ HSC,^[^ ^75^ ^]^ NIH3T3,^[^ ^76^ ^]^ HEK293,^[^ ^76^ ^]^ MDCK,^[^ ^76^ ^]^ HeLa,^[^ ^76^ ^]^ HCT116^[^ ^76^ ^]^	FL‐dextran,^[^ ^75^, ^76^ ^]^ Alexa488‐protein^[^ ^76^ ^]^	RFP pDNA,^[^ ^75^ ^]^ Cas9 RNP,^[^ ^75^ ^]^ siRNA^[^ ^76^ ^]^	10^3^ cells min^−1^,^[^ ^75^ ^]^ 10^5^ cells min^−1[^ ^76^ ^]^	–
		Static poking	Jurkat,^[^ ^77^ ^]^ K562,^[^ ^77^ ^]^ human T cell ^[^ ^77^ ^]^	PI dye^[^ ^77^ ^]^	GFP pDNA^[^ ^77^ ^]^	10^4^ cells per run^[^ ^77^ ^]^	Under commercialization by Basilard Biotech^[^ ^77^ ^]^
		Hydrodynamic cell stretching	A2780cis,^[^ ^78^ ^]^ ES2,^[^ ^78^ ^]^ HEK293,^[^ ^78^ ^]^ MDA231,^[^ ^15^, ^78^ ^]^ K562,^[^ ^15^, ^78^ ^]^ HEK293t,^[^ ^15^ ^]^ 3T3,^[^ ^15^ ^]^ DC 2.4,^[^ ^15^ ^]^ HeLa,^[^ ^15^ ^]^ human MSC,^[^ ^15^ ^]^ human ADSC,^[^ ^15^ ^]^ murine BMDC ^[^ ^15^ ^]^	FITC–dextran^[^ ^15^, ^78^ ^]^	DNA nanostructure,^[^ ^78^ ^]^ CRISPR/Cas9,^[^ ^78^ ^]^ siRNA,^[^ ^15^, ^78^ ^]^ GFP pDNA,^[^ ^15^, ^78^ ^]^ GFP mRNA,^[^ ^15^ ^]^ Qdot,^[^ ^15^ ^]^ nanobead^[^ ^15^ ^]^	10^6^ cells min^−1[^ ^15^, ^78^ ^]^	Demonstrated 300 nm nanoparticle delivery;^[^ ^15^ ^]^ under commercialization by MxT Biotech^[^ ^15^ ^]^
	Microinjection	Microinjection on chip	HeLa,^[^ ^84^, ^90^ ^]^ zebrafish embryo,^[^ ^83^ ^]^ *Drosophila* embryo^[^ ^91^, ^93^ ^]^	FL‐dextran,^[^ ^84^, ^90^ ^]^ methylene blue,^[^ ^83^ ^]^ rhodamine B,^[^ ^83^, ^93^ ^]^ fluorescein,^[^ ^91^ ^]^ NaN_3_^[^ ^93^ ^]^	siRNA^[^ ^91^ ^]^	60 cells min^−1[^ ^84^ ^]^	–
		Probe tip injection	C2C12,^[^ ^186^ ^]^ NG108,^[^ ^186^ ^]^ MCF7,^[^ ^187^ ^]^ RAW 264.7,^[^ ^187^ ^]^ RKO^[^ ^187^ ^]^	FITC‐sodium salt,^[^ ^186^ ^]^ CellTracker green^[^ ^186^ ^]^	FITC‐nanodiamond^[^ ^187^ ^]^	N/A	Commercialized by Cytosurge AG^[^ ^186^ ^]^
	Acoustic cavitation	Ultrasonic wave	H9C2^[^ ^111^ ^]^	FITC‐dextran^[^ ^111^ ^]^	Pharmaceutical agents (doxorubicin, apigenin, and luteolin)^[^ ^111^ ^]^	N/A	No microbubble required^[^ ^111^ ^]^
		Bubble oscillation	HUVEC,^[^ ^114^ ^]^ primary rat aortic‐smooth muscle cell ^[^ ^99^ ^]^	DOX‐liposome,^[^ ^114^ ^]^ calcein^[^ ^99^ ^]^	N/A	N/A	Vessels‐on‐a‐chip cytotoxic drug delivery^[^ ^114^ ^]^
		Surface acoustic wave	MCF7^[^ ^115^ ^]^	PI dye^[^ ^115^ ^]^	N/A	N/A	–
		Acoustic wave cell manipulation	Jurkat,^[^ ^117^ ^]^ MEF,^[^ ^117^ ^]^ PBMC,^[^ ^117^ ^]^ HSPC ^[^ ^117^ ^]^	Cy3‐labeled DNA^[^ ^117^ ^]^	GFP pDNA^[^ ^117^ ^]^	10^5^ cells min^−1[^ ^117^ ^]^	Not requiring microbubble^[^ ^117^ ^]^
	Laser cavitation	Single bubble cavitation	HL60,^[^ ^118^ ^]^ myeloma cell^[^ ^119^ ^]^	Trypan blue^[^ ^118^, ^119^ ^]^	N/A	N/A	–
		Microbubble array cavitation	HeLa,^[^ ^120^ ^]^ PB‐MDMs,^[^ ^120^ ^]^ NHDFs,^[^ ^120^ ^]^ RPTECs ^[^ ^120^ ^]^	FITC–dextran^[^ ^120^ ^]^	Antibody,^[^ ^120^ ^]^ enzyme,^[^ ^120^ ^]^ bacteria,^[^ ^120^ ^]^ nanobead^[^ ^120^ ^]^	10^5^ cells min^−1[^ ^120^ ^]^	Macromolecule delivery^[^ ^120^ ^]^
Electroporation	Cell trapping‐based electroporation	Microhole trapping	ND‐1^[^ ^149^, ^150^ ^]^	YOYO‐1 dye^[^ ^150^ ^]^	EGFP pDNA^[^ ^150^ ^]^	N/A	–
		Channel trapping	HeLa,^[^ ^151^, ^152^, ^153^ ^]^ C2C12,^[^ ^155^ ^]^ human MSC ^[^ ^155^ ^]^	Trypan blue,^[^ ^151^ ^]^ calcein,^[^ ^151^, ^152^, ^153^ ^]^ FL‐dextran,^[^ ^152^, ^153^ ^]^ PI dye^[^ ^155^ ^]^	EGFP‐ERK1 pDNA^[^ ^155^ ^]^	N/A	–
		Membrane sandwich electroporation	NIH 3T3,^[^ ^156^ ^]^ mouse ES ^[^ ^157^, ^158^ ^]^	N/A	GFP pDNA,^[^ ^156^, ^157^ ^]^ SEAP pDNA^[^ ^156^, ^157^, ^158^ ^]^	10^4^ cells per run^[^ ^157^ ^]^	–
	Nanochannel electroporation	Single‐cell electroporation	Jurkat,^[^ ^163^ ^]^ K562,^[^ ^163^ ^]^ mouse embryonic fibroblasts ^[^ ^163^ ^]^	PI dye,^[^ ^163^ ^]^ Cy3‐ODN^[^ ^163^ ^]^	GAPDH molecular beacon,^[^ ^163^ ^]^ siRNA,^[^ ^163^ ^]^ Qdot,^[^ ^163^ ^]^ Cy3‐labeled GFP pDNA^[^ ^163^ ^]^	N/A	Faster delivery compared with bulk and microelectroporation^[^ ^163^ ^]^
		Nanochannel array electroporation	NK‐92,^[^ ^166^ ^]^ H9C2,^[^ ^166^, ^167^ ^]^ MEF,^[^ ^165^, ^167^ ^]^ mouse cardiomyocyte,^[^ ^165^ ^]^ MDA231,^[^ ^168^ ^]^ CHO,^[^ ^168^ ^]^ HT1080^[^ ^168^ ^]^	PI dye,^[^ ^166^, ^167^, ^168^ ^]^ FAM‐ODN,^[^ ^167^ ^]^ Co^2+^,^[^ ^168^ ^]^ Alexa488‐BSA^[^ ^168^ ^]^	CAR/GFP pDNA,^[^ ^166^ ^]^ GFP pDNA,^[^ ^165^ ^]^ OSKM pDNA,^[^ ^167^ ^]^ miRNA,^[^ ^165^ ^]^ mCherry pDNA^[^ ^168^ ^]^	10^4^ cells per cm^2^,^[^ ^167^ ^]^ 10^6^ cells per run^[^ ^169^ ^]^	mRNA internalization into exosome^[^ ^169^ ^]^
		Nanopore electroporation	HeLa,^[^ ^170^ ^]^ HEK293,^[^ ^170^ ^]^ 3T3,^[^ ^170^ ^]^ Jurkat^[^ ^170^ ^]^	N/A	mCherry mRNA,^[^ ^170^ ^]^ GFP pDNA^[^ ^170^ ^]^	10^4^ cells per run^[^ ^170^ ^]^	–
		Nanoinjection	Human MSC ^[^ ^171^ ^]^	RFP^[^ ^171^ ^]^	GFP pDNA^[^ ^171^ ^]^	N/A	Under commercialization by Femtobiomed^[^ ^171^ ^]^
		Nanostraw electroporation	CHO,^[^ ^174^ ^]^ HEK293t,^[^ ^174^ ^]^ HEK293,^[^ ^176^ ^]^ hiPSC‐CMs,^[^ ^176^ ^]^ HSCs,^[^ ^176^ ^]^ HFs,^[^ ^176^ ^]^ mouse glia,^[^ ^176^ ^]^ mouse neuron,^[^ ^176^ ^]^ HSPCs ^[^ ^142^ ^]^	PI dye,^[^ ^174^ ^]^ FL‐dextran,^[^ ^142^ ^]^ FITC‐OGN^[^ ^142^ ^]^	RFP pDNA,^[^ ^174^ ^]^ mCherry mRNA,^[^ ^176^ ^]^ GFP mRNA,^[^ ^142^, ^176^ ^]^ STIM1 protein,^[^ ^176^ ^]^ Cas9 RNP,^[^ ^176^ ^]^ siRNA^[^ ^142^ ^]^	10^5^ cells per run^[^ ^176^ ^]^	Under commercialization by NAVAN Technologies^[^ ^174^ ^]^
	Nanochannel electroporation	Nanofountain electroporation	HeLa,^[^ ^181^, ^182^ ^]^ HT1080,^[^ ^182^ ^]^ HEK293^[^ ^183^ ^]^	FL‐dextran,^[^ ^181^, ^182^ ^]^ BSA^[^ ^181^, ^182^, ^183^ ^]^	GAPDH‐molecular beacon,^[^ ^181^, ^182^ ^]^ GFP pDNA,^[^ ^181^, ^183^ ^]^ GFP knockdown CRISPR/Cas9^[^ ^183^ ^]^	N/A	–
	Flow‐through electroporation	Flow‐through DC electroporation	CHO,^[^ ^189^, ^190^, ^191^, ^196^ ^]^ K562^[^ ^197^ ^]^	SYTOX dye,^[^ ^189^, ^190^ ^]^ PI dye^[^ ^197^ ^]^	GFP pDNA,^[^ ^190^, ^191^, ^197^ ^]^ Qdot^[^ ^196^ ^]^	10^5^ cells min^−1^,^[^ ^197^ ^]^ 10^6^ cells min^−1^,^[^ ^190^ ^]^ 10^7^ cells min^−1[^ ^191^ ^]^	–
		Flow‐through AC electroporation	Huh‐7,^[^ ^188^ ^]^ K562,^[^ ^198^ ^]^ CT26.WT,^[^ ^198^ ^]^ C3H10T1/2,^[^ ^199^ ^]^ HeLa,^[^ ^200^ ^]^ CHO,^[^ ^193^ ^]^ HEK293a,^[^ ^201^ ^]^ Neuro‐2a,^[^ ^201^ ^]^ HUVEC,^[^ ^201^ ^]^ HSF ^[^ ^201^ ^]^	PI dye,^[^ ^198^ ^]^ FL‐dextran,^[^ ^200^ ^]^ phallotoxin^[^ ^200^ ^]^	*β*‐galactosidase pDNA,^[^ ^188^ ^]^ GFP pDNA,^[^ ^188^, ^193^, ^198^, ^199^, ^201^ ^]^ GFP siRNA^[^ ^200^ ^]^	10^3^ cells min^−1^,^[^ ^199^ ^]^ 10^4^ cells min^−1^,^[^ ^201^ ^]^ 10^6^ cells min^−1[^ ^198^ ^]^	–
		Flow electroporation with laminar flow	Yeast cell,^[^ ^202^ ^]^ HEK293a,^[^ ^203^ ^]^ HEK293,^[^ ^203^ ^]^ HeLa,^[^ ^203^ ^]^ C2C12,^[^ ^203^ ^]^ Neuro‐2a,^[^ ^203^ ^]^ PC12,^[^ ^203^ ^]^ human T cell ^[^ ^205^, ^206^ ^]^	Fluorescein^[^ ^202^ ^]^	siRNA,^[^ ^203^ ^]^ GFP pDNA,^[^ ^203^ ^]^ mCherry mRNA^[^ ^205^, ^206^ ^]^	10^4^ cells min^−1^,^[^ ^202^ ^]^ 10^5^ cells min^−1^,^[^ ^203^, ^205^ ^]^ 10^7^ cells min^−1[^ ^206^ ^]^	Electroporation assisted by acoustophoretic cell manipulation^[^ ^205^ ^]^
		Flow electroporation with Dean/vortex flow	CHO,^[^ ^204^ ^]^ MDA231,^[^ ^207^, ^208^, ^210^ ^]^ HEK293,^[^ ^208^ ^]^ MCF7,^[^ ^208^ ^]^ HeLa^[^ ^208^ ^]^	YOYO‐1 dye,^[^ ^204^ ^]^ PI dye,^[^ ^208^, ^210^ ^]^ FL‐protein^[^ ^208^ ^]^	GFP pDNA,^[^ ^204^, ^210^ ^]^ RFP pDNA,^[^ ^210^ ^]^ drug (gemcitabine, bleomycin, topotecan, and quercetin),^[^ ^207^ ^]^ siRNA,^[^ ^208^ ^]^ miRNA^[^ ^208^ ^]^	10^5^ cells min^−1[^ ^204^ ^]^	Cytotoxic drug delivery^[^ ^207^ ^]^
	Droplet electroporation	Flow‐through droplet electroporation	Yeast cell,^[^ ^212^ ^]^ CHO,^[^ ^213^ ^]^ green microalgae (*C. reinhardtii*)^[^ ^214^ ^]^	Fluorescein,^[^ ^212^ ^]^ SYTOX green dye^[^ ^213^ ^]^	GFP pDNA,^[^ ^213^ ^]^ DNA fragment^[^ ^214^ ^]^	10^4^ cells min^−1[^ ^213^ ^]^	Delivery assisted by Dean vortices^[^ ^214^ ^]^
		EWOD droplet electroporation	*E. coli* ^[^ ^216^ ^]^	N/A	Antibiotic resistance pDNA^[^ ^216^ ^]^	N/A	Transfection efficiency assessed using colony forming unit^[^ ^216^ ^]^

The third point for consideration is the necessity of cell functional studies after delivery. Many studies have mainly focused on reporting delivery efficiency and cell viability as key metrics (note that a standardized method characterizing delivery efficiency and viability should be set and used by the community for an unbiased comparison). However, functional studies should be included for cells treated with the developed method. Functional studies include genome‐wide expression profiling, long‐lasting functionality analysis, and investigation of post‐treatment mortality, long‐term cell proliferation, and in vivo therapeutic efficacy. Recently, DiTommaso et al. found no statistical difference between cells treated with electroporation and mechanoporation with regard to delivery efficiency and cell viability. However, substantial dysregulation of key genes, functional pathways, and disease markers was observed only in cells treated with electroporation.^[^ ^57^ ^]^ In addition, in vivo therapeutic efficacy (e.g., ineffective tumor reduction from electroporation) differences were observed, depending on the delivery method. Therefore, functional level characterizations, in addition to delivery performance, should also be carefully investigated.

The fourth consideration is cGMP compliance of the developed intracellular delivery method. cGMP, enforced by the FDA, provides regulations assuring proper design, monitoring, and control of manufacturing processes and facilities. cGMP regulations require establishing strong quality management systems, obtaining appropriate quality materials, establishing robust operation, detecting and investigating product quality deviations, and maintaining reliable testing laboratories (FDA cGMP regulations for drugs). One of the overarching goals of developing a novel intracellular delivery platform is for it to be used for clinical applications; thus, cGMP considerations should be made as early as possible. Particularly, reliable and robust delivery, delivery process monitoring, delivery in a sterile condition/environment, minimized human intervention, and process standardization and scalability are key features toward future cGMP compliance.^[^ ^217^ ^]^

Finally, commercialization efforts should be continued in parallel. Bulk electroporation has been successfully commercialized (e.g., Lonza's Nucleofector, Mirus’ Ingenio EZporator, and Bio‐Rad's Gene Pulser Xcell System) and capillary electroporation‐based products (e.g., Invitrogen's Neon transfection system) are also available in the market. Encouragingly, the future appears bright as several startup companies, including SQZ Biotech, Kytopen, Indee Labs, CellFe, NAVAN Technologies, Basilard BioTech, Femtobiomed, and MxT Biotech, based on microfluidic developments have recently been launched to develop new methods for cell‐based therapies. Big pharmaceutical companies are also involved in this process; for instance, Roche signed a $1 billion deal with SQZ Biotech focusing on antigen‐presenting cell (APC)‐based product developments.^[^ ^218^ ^]^ This effort is crucial because this is how the value proposition and potential of a microfluidic solution are developed in the field or laboratory.

In summary, although there are many challenges ahead, significant progress has been achieved via nanofluidic and microfluidic intracellular delivery. Together with new opportunities from cell‐based therapies, growing attention, and ongoing commercialization efforts, microfluidic and nanofluidic intracellular delivery hold the potential to be a next‐generation intracellular delivery platform through continued development and optimization. We envision that micro‐ and nanofluidic intracellular delivery methods will critically influence cellular engineering and cell therapy fields in the near future.

## Conflict of Interest

A.J.C. has an equity interest in MxT Biotech, which is a commercializing microfluidic intracellular delivery technology that is described herein.
